# An AI-based module for interstitial glucose forecasting enabling a “Do-It-Yourself” application for people with type 1 diabetes

**DOI:** 10.3389/fdgth.2025.1534830

**Published:** 2025-06-13

**Authors:** Antonio J. Rodriguez-Almeida, Guillermo V. Socorro-Marrero, Carmelo Betancort, Garlene Zamora-Zamorano, Alejandro Deniz-Garcia, María L. Álvarez-Malé, Eirik Årsand, Cristina Soguero-Ruiz, Ana M. Wägner, Conceição Granja, Gustavo M. Callico, Himar Fabelo

**Affiliations:** ^1^ Institute for Applied Microelectronics, University of Las Palmas de Gran Canaria, ULPGC, Las Palmas de Gran Canaria, Spain; ^2^ Endocrinology and Nutrition Department, Complejo Hospitalario Universitario Insular Materno-Infantil, CHUIMI, Las Palmas de Gran Canaria, Spain; ^3^ Instituto de Investigaciones Biomédicas y Sanitarias, University of de Las Palmas de Gran Canaria, ULPGC, Las Palmas de Gran Canaria, Spain; ^4^ Department of Computer Science, University of Tromsø—The Arctic University of Norway, Tromsø, Norway; ^5^ Norwegian Centre for E-health Research, University Hospital of North-Norway, UNN, Tromsø, Norway; ^6^ Department of Signal Theory and Communications, Telematics and Computing Systems, Rey Juan Carlos University, URJC, Fuenlabrada, Spain; ^7^ Faculty of Nursing and Health Sciences, Nord University, NU, Bodø, Norway; ^8^ Fundación Canaria Instituto de Investigación Sanitaria de Canarias, FIISC, Las Palmas de Gran Canaria, Spain; ^9^ Research Unit, Hospital Universitario de Gran Canaria Dr. Negrin, Las Palmas de Gran Canaria, Spain

**Keywords:** type 1 diabetes, deep learning, personalized medicine, continuous glucose monitoring, mHealth

## Abstract

**Introduction:**

Diabetes mellitus (DM) is a chronic condition defined by increased blood glucose that affects more than 500 million adults. Type 1 diabetes (T1D) needs to be treated with insulin. Keeping glucose within the desired range is challenging. Despite the advances in the mHealth field, the appearance of the do-it-yourself (DIY) tools, and the progress in glucose level prediction based on deep learning (DL), these tools fail to engage the users in the long-term. This limits the benefits that they could have on the daily T1D self-management, specifically by providing an accurate prediction of their short-term glucose level.

**Methods:**

This work proposed a DL-based DIY framework for interstitial glucose prediction using continuous glucose monitoring (CGM) data to generate one personalized DL model per user, without using data from other people. The DIY module reads the CGM raw data (as it would be uploaded by the potential users of this tool), and automatically prepares them to train and validate a DL model to perform glucose predictions up to one hour ahead. For training and validation, 1 year of CGM data collected from 29 subjects with T1D were used.

**Results and Discussion:**

Results showed prediction performance comparable to the state-of-the-art, using only CGM data. To the best of our knowledge, this work is the first one in providing a DL-based DIY approach for fully personalized glucose prediction. Moreover, this framework is open source and has been deployed in Docker, enabling its standalone use, its integration on a smartphone application, or the experimentation with novel DL architectures.

## 1 Introduction

Diabetes Mellitus (DM) is a chronic metabolic condition characterized by increased blood glucose level concentrations, which was suffered by around 537 million adults in 2021, representing nearly 10% of the world population (1). Moreover, the number of people with diabetes will potentially grow to 643 million by 2030, with an estimation of over 6.7 million deaths from diabetes-related issues (1, 2). Chronic hyperglycemia may lead to DM-associated complications, including damage to the heart, eyes, kidneys, blood vessels and nerves, seriously affecting the quality of life of those who suffer it (3). The two main types of DM are type 2 diabetes, caused by an increased resistance to insulin, where the body is not able to secrete enough insulin to overcome such resistance, and Type 1 Diabetes (T1D), which results from autoimmune, pancreatic beta-cell destructions that ends in a complete lack of insulin production (3, 4).

Particularly, T1D is the main type of diabetes in childhood, although it can occur at any point in life. It accounts for 8.75 million people globally, 1.52 million of them being less than 20 years old by year 2021 (1). T1D treatment is a challenge, since imperfect, exogenous insulin administration aims to mimic (inexistent) endogenous insulin production, to keep glucose concentrations within a safe range, avoiding hyper- and hypoglycemia (high and low blood glucose levels, respectively). An individual with T1D experiences around two episodes of symptomatic hypoglycemia per week, which is directly associated with an increase in morbidity and mortality (5, 6). Due to the threat that these recurrent hypoglycemic events entail, T1D requires a lifetime of thorough self-management. To maintain the blood glucose levels in an appropriate range, people with T1D require daily administration of insulin, otherwise their lives would be seriously endangered. This control, together with regular consultations with an endocrinologist, are crucial to achieve good metabolic control, being able to live a healthy life and potentially mitigating the hardest long-term complications of T1D (3, 7).

T1D self-management mainly includes glucose monitoring and subcutaneous insulin administration. Current glucose monitoring tools can be classified in two groups: capillary blood glucose measurements, which requires a puncture in a finger to extract a blood drop with a glucometer, and Continuous interstitial Glucose Monitoring (CGM), a technique that has become standard of care in many countries, and whose cost decrease and accuracy improvements will likely contribute to its expansion in the short-term (8). CGM devices use a sensor attached to the subject's skin that monitors interstitial glucose concentrations (typically every 5 or 15 min, depending on the model of the sensor) (9). Supplementary Figure S1a depicts a segment of a CGM signal. There is an international consensus to establish the guidance for assessment glycemic control taking the CGM as reference, although glucometers are still more accurate than CGM sensors. The target glucose range is 70–180 mg/dl, and the time spent within these values is called Time In Range (TIR). TIR is recommended to be greater than 70% daily. The Time Above Range (TAR), i.e., with glucose concentrations over 180 mg/dl is advisable to be less than 25%, whereas the Time Below Range (TBR), should not be more than 4% of the day, corresponding to less than one hour (10). Therefore, the T1D individual's goal is to have the highest TIR possible, decreasing the TAR and TBR as much as possible. The visualization standard of these ranges in T1D dedicated mobile applications is illustrated in Supplementary Figure S1b.

Current functionalities for the available commercialized CGM devices are the glucose data transfer to a display device (e.g., a smartphone), sometimes triggering alarms about the probable arise of hyper- or hypoglycemia episodes (9). CGM has proven to reduce the number of hypoglycemic events in people with T1D (11). Besides, continuous glucose sensors have also enabled the communication of the glucose readings to the insulin pumps, giving rise to the so-called hybrid closed loop systems, where basal and correction insulin infusions are automated, but the mealtime insulin is not, since there are no commercially available systems that provide meal information automatically (9).

Lately, mobile Health (mHealth) applications have been increasingly employed to enhance chronic conditions management, which is crucial to improve the individual's health outcomes (12). However, they have failed to address the challenge of individual's long-term non-adherence to the tools themselves, due mainly to data privacy concerns, lack of perceived usefulness, non-optimal usability and the potential costs associated to their use (13). Seeking engagement in mHealth applications, and to overcome the bureaucratic delays associated with regulatory processes, the *Do-It-Yourself* (DIY) approach has gained popularity recently (2). This approach facilitates the self-management of an individual's condition by easy access to their health data. In the diabetes field, the turning point arrived in 2014 with the *Nightscout Project*, the first reported DIY mHealth application for people with diabetes (14). In 2022, Morrison et al. published a review of DIY systems used in people with T1D, evidencing that people are willing to use this kind of systems rather than the commercial ones (15). Additionally, in this approach the individuals are the digital tool providers, instead of medical companies, thus enabling an easy-to-use and subject-driven approach (16), which is strongly related with the tool engagement and usability (17). Furthermore, DIY systems have demonstrated to be effective in the glycemic control in people with diabetes (18). Although in the diabetes context DIY refers to people with this condition developing the tool by themselves, in this work this term refers to their indirect participation through the co-creation process of this module, the fact that this open source module allows individuals to generate their own personalized models using their data, together with the fact that researchers and/or software developers could implement further improvements on it, and implement it as an smartphone application.

Summarizing, despite the outlined advances in glucose monitoring, most people with T1D do not reach optimal glycemic control (19). Although there are diverse reasons that explain this, the most relevant factors that could be mitigated by the implementation of a personalized AI-based tool are:

a. The high intra-subject variability in CGM measurements in people with T1D, the errors in glucose measurements and the delay in the control actions (20).
b. The suboptimal usability of the current available digital tools for T1D management, that ends on the disregard of many individuals for them (17).
c. The lack of personalization in the available digital tools that could provide accurate and customized feedback, considering individual metabolism, environment, or digital alphabetization (2).

An accurate and reliable prediction of the glucose concentration in the next minutes or hours would certainly support individuals, anticipating possible hyper- and hypoglycemic events. This knowledge is already included in the hybrid close-loop systems to improve the insulin delivery (21). There are numerous studies that have assessed this task using mainly three approaches. Physiological models use equations to describe human metabolism; data-driven models, where this work is enclosed, are based on time series analysis and Machine/Deep learning (ML/DL) techniques; and hybrid models are a combination of the above (22, 23). The data-driven models are the ones that have presented the best prediction performance so far (22). Most of the studies, including those with the most accurate predictions, are based on Convolutional Neural Networks (CNNs) (24, 25) and Long Short-Term Memory (LSTM) architectures (26). However, although the advances in this field are promising, most of the published literature lacks a subject-oriented approach in the Artificial Intelligence (AI) implementation and evaluation, limiting the impact that these algorithms can have on people's lives.

**Figure 1:**
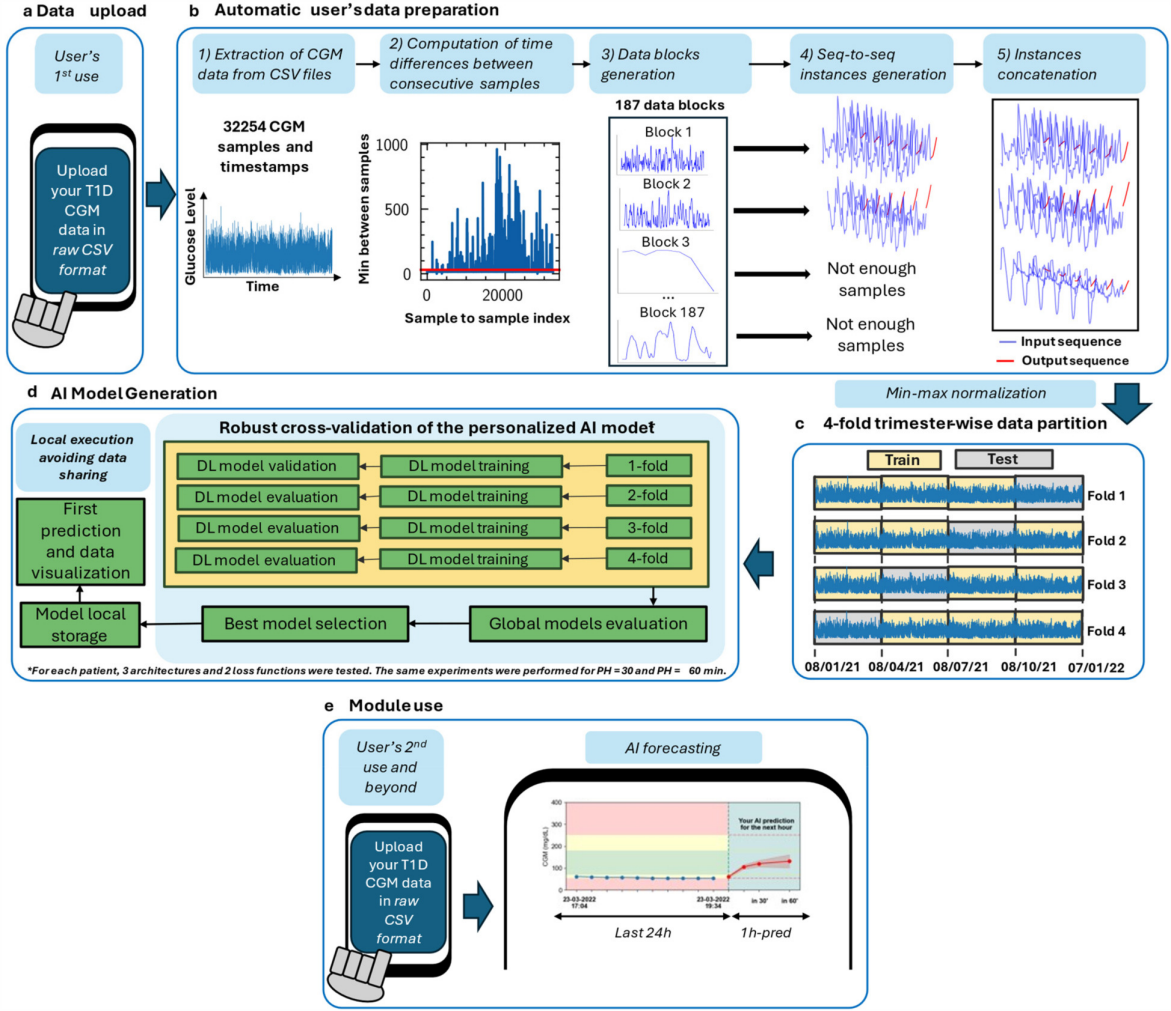
Proposed AI-based DIY framework for interstitial glucose level prediction up to 30 and 60 min in people with T1D. Framework steps are illustrated in order. The zone delimited by dashed lines is only executed once: in the user's first use. Out of this zone, the steps that the user will perform every time he/she uses the DIY module are illustrated. For the analysis presented in this work, this framework has been executed once per included subject (*n* = 29). After the first use, the module is designed to, every time the user “triggers” it, automatically read the last day of the available CGM data and provide an immediate 30- or 60-min prediction. Currently, if there are CGM readings interruptions, a prediction is not provided. **(a)** In the first use, the users upload the raw CGM data to generate the DL model as data were downloaded by the corresponding application. In subsequent uses, user allows the Reading of the last day of data to perform a prediction. **(b)** Automatic data analysis and preparation is performed to check if the provided data allows a reliable AI model generation or not. If so, data is prepared to train and validate the model. **(c)** Min-max normalization and 4-fold trimester-wise data partition. **(d)** AI model generation after fold shuffling, train, validation, model evaluation, and best model selection. **(e)** Illustration of subsequent module uses, including interstitial glucose prediction and data visualization.

Currently, there is a scenario where the arrival of DIY tools embraced by people with T1D, the promising advances of AI in glucose forecasting, and the progress in the mHealth field do not fully converge, thus limiting the implementation of such systems in the healthcare programs (27). Additionally, data privacy has become one of the main concerns of the users when using personal and clinical data in mobile applications (28), which is related to the reservations of using them daily (29). Thus, the proposed AI-based DIY framework (Figure 1) intends to merge these advances and overcome these limitations, contributing in terms of AI model personalization and data privacy to enhance T1D self-management including the following features:

a. Robust validation approach of fully personalized AI-based models (using only the user's own data) to make 30- and 60-min interstitial glucose forecasting, based on the state-of-the-art architectures using 1 year of CGM data.
b. Integration of the DL workflow in a DIY module for software developers that includes personalization in the way T1D data is shown to the user.
c. AI-related information, targeting user empowerment in the use of AI, informing them about the relationship between their data and the AI models generated (e.g., why a DL model cannot be reliably trained with the provided data) (30).
d. Secure design that enables the local generation and execution of the DL model in the user's own device, avoiding sharing personal data with third parties.
e. Open-source modular design that allows researchers and developers the integration of data from new sensors, preprocessing stages, or additional DL architectures in a straightforward way.

To the best of our knowledge, this work proposes the first personalized AI-based DIY module for both software developers and people with T1D to aid the latter with their daily self-management by providing up to one-hour interstitial glucose level forecasting. The design of this module allows its easy integration on a smartphone, even if it only has been tested on a computer. For the sake of usability improvement and user engagement, this tool has been designed considering the daily habits of people with T1D regarding their glycemic control, and the challenges they usually face through a co-creation process. Besides, this has been achieved following a robust methodology to train and technically validate the DL models, using 1 year of CGM data, and a global and subject-wise evaluation. This module automatically analyses the individual's data, trains, and validates a fully personalized DL model once the user has uploaded her/his CGM data. As a result, the user obtains a detailed visualization of their data in a standardized but customizable manner, as well as a forecast of her/his interstitial glucose level. If a hypo- or hyperglycemic episode is predicted, a warning message will be triggered.

## 2 Materials and methods

### 2.1 T1D dataset

CGM data were collected at the *Complejo Hospitalario Insular-Materno Infantil de Las Palmas de Gran Canaria* from 41 people with T1D that use a sensor from the FreeStyle Libre (Abbott) family. Additional data, such as physical activity, insulin administration, or carbohydrates, were not available for any of the subjects involved in this study. The raw data were extracted from the LibreView^TM^ (Abbott) website by an endocrinologist in raw Comma-Separated Values (CSV) format. Participants were invited to participate and were given oral and written information. All participants signed a written informed consent form. Individual's data were stored on a secure server only accessible through a virtual private network by people working on the WARIFA project (31) after being given individual access credentials.

Supplementary Table S2 summarizes each subject's more relevant information regarding AI processing associated with an anonymous ID. To train DL models, a sufficient number of samples for each subject is needed. Among the 41 subjects there were cases in which this requirement was not fulfilled, due to sensor malfunction or due to multiple sensor replacements in a short period of time. Moreover, different sampling periods in the sensors could imply variations in the considered architectures. Thus, only the subjects that met the following inclusion criteria were considered in this study:

1. To have at least 1 year of CGM readings with the same sensor. Sensor malfunction (e.g., reading interruptions) was considered and further treated to conform the instances to train and validate the DL models.
2. To have a glucose sensor with a sampling period of 15 min, aiming a fair prediction performance comparison between different subjects.

Finally, 29 subjects met the inclusion criteria, for whom it was used the oldest year (when more available) of these subjects to train and validate the personalized AI-models. The oldest year was selected so subsequent CGM readings were left for potential further testing of the DL models. Besides, when available, additional data were collected for a final model test.

### 2.2 CGM data preparation and preprocessing

The data curation phase was performed by filtering out the subjects that did not meet the inclusion criteria previously described. The framework was executed one time per included subject (i.e., 29 times) using the first recorded year of each subject. At this point, there was a unique sequence of CGM readings for each subject with their associated timestamps to which a pre-processing step was applied. At that point, data were still not suitable for the training of the DL models.

Then, an automatic and parametric subject-per-subject sequence-to-sequence (from now, *seq-to-seq*) (i.e., an input sequence and an output sequence) dataset generation was developed (Figure 1B). The considered parameters were the *window length*, denoted as *N* (96 in this work), the *step* from one sequence to the next one (set to one in this case), and *prediction steps*, which is the result of the division between the Prediction Horizon (PH), i.e., how far ahead the model predicts, and the sensor sampling period. For this case, this parameter was set to four and two (60- and 30-min PH/15 min sampling period, respectively). Notice that, with this approach, there is a trade-off between the window input length and number of training instances; a larger input length will likely provide more information to the DL model, but fewer training instances will be generated, potentially limiting the prediction performance.

Firstly, the time intervals between two consecutive readings were computed considering the sensor timestamps associated with all readings. Although the nominal sampling period of all the sensors included in this work is 15 min, the actual interval between consecutive samples may vary from this reference value (Supplementary Figure S10). Therefore, to be compliant with the assumption of data provided in periodic time slots, two samples are actually consecutive when the time between them is lower than twice the sampling period (i.e., 30 min). As depicted in Supplementary Figure S6, it is assumed that, if the sensor reading is delayed or advanced in 2 × sampling period minus 1 s (i.e., 29:59 min), that reading corresponds to the next timeslot.

Every time that two consecutive samples in the CGM sequence surpassed 29:59 min, a new data block was created. Therefore, the number of blocks depends on the number and location of interruptions in the data (Figure 1B). Furthermore, the minimum number of consecutive samples to form a usable block (i.e., to form an instance suitable to train the DL models) is equal to the length of the input sequence plus the length of the output sequence. Otherwise, those samples are discarded and will not be used to generate and train the AI models. Notice that the ideal case would be to have only one data block without interruptions. After the data block generation, each data block is swept into steps of one to generate the model inputs (*X*) and the associated outputs (*Y*). Thus, block length determines the number of instances a particular block provides. After the sequences were generated from all data blocks, they are concatenated as instances to perform the min-max normalization as described in Equation 1.

(1) $$i_{norm}=\frac{i-S_{min}}{S_{max}-\;S_{min}},$$

where *i* represents a given sample $i_{norm}$ is the normalized sample, and $S_{max}$ and $S_{min}$ the global maximum and minimum values of a subject's data.

Additionally, there were a few cases where the CSV file did not follow LibreView's standard way of presenting the data. In such cases, glucose concentration values were considered corrupted, and thus treated as interruptions.

### 2.3 ISO adapted loss function

ISO 15197:2015 (32) establishes two specific criteria for glucose concentration monitoring systems, both based on the discrepancy between measured values and their reference counterparts. Although this standard was developed for glucose sensor measurement errors, its criteria can be extrapolated to evaluate errors between the actual glucose concentration and a value predicted by a model, tailoring the prediction problem assessment to the glucose monitoring context. AI-based models, such as those proposed in this work, are often trained with the aim of minimizing standard loss functions based on squared or absolute errors. Although theoretically both approaches lead to the same optimal solution, in practice a better result is expected if the loss functions are adapted to the required validation performance. A customized loss function has therefore been designed to enforce compliance with the criteria specified in the ISO standard.

Firstly, the two validation criteria, i.e., the one referring to the acceptable error zone in terms of accuracy and the one attending to regions A and B in the Consensus Error Grid [CEG—also called Parkes Error Grid (PEG)] (24, 26), have been analyzed by comparing the maximum admissible error values for each case. As shown in Supplementary Figure S7, the former criterion is the most restrictive except in the narrow range of reference values from 164 to 174 mg/dl, where the maximum admissible error is established by the boundary of region A in the CEG. Hence, it could be assumed that error values of 15 mg/dl or 15% of the reference value, depending on whether the actual concentration is less than 100 mg/dl or not, respectively, define the upper bound of the admissible error.

The strategy for designing an error function explicitly considering this upper bound is to force a barrier in the form of a steep slope of the function in the neighborhood of the threshold value. This can be achieved by introducing an additive term to the quadratic error that can be considered negligible (ideally zero) in the admissible region but large (ideally infinite) elsewhere. Nevertheless, the desirable properties of the error function must be preserved to facilitate the optimization process of AI model training, specifically its increasing monotonicity with the magnitude of the error and its smoothness. It is inspired by the design of low-pass filters, where the constraints are similar, with a low attenuation in the admissible region and a cut-off value beyond which the attenuation is high. Therefore, the additive term γ shown in the following Equation 2 is proposed:

(2) $$\gamma (\epsilon )=K\epsilon^{2n}$$

where the variable ϵ refers to the normalised error in the prediction of a sample, i.e., the error itself if less than 100 mg/dl or the error multiplied by $\;\frac{100}{r}$, being *r* the reference value associated with that sample, and *K* is the proper constant to conform the transition in a way that the term remains below a prescribed tolerance in the admissible region and surpasses an established threshold for large error values.

The overall expression for the error function ($L_{ISO}$), including the quadratic error and the additive term γ is detailed in Equation 3.

(3) $$L_{ISO}(\epsilon )=\;\epsilon^{2}+\gamma (\epsilon )=\;\epsilon^{2}+K\;\epsilon^{2n}$$

**Figure 2:**
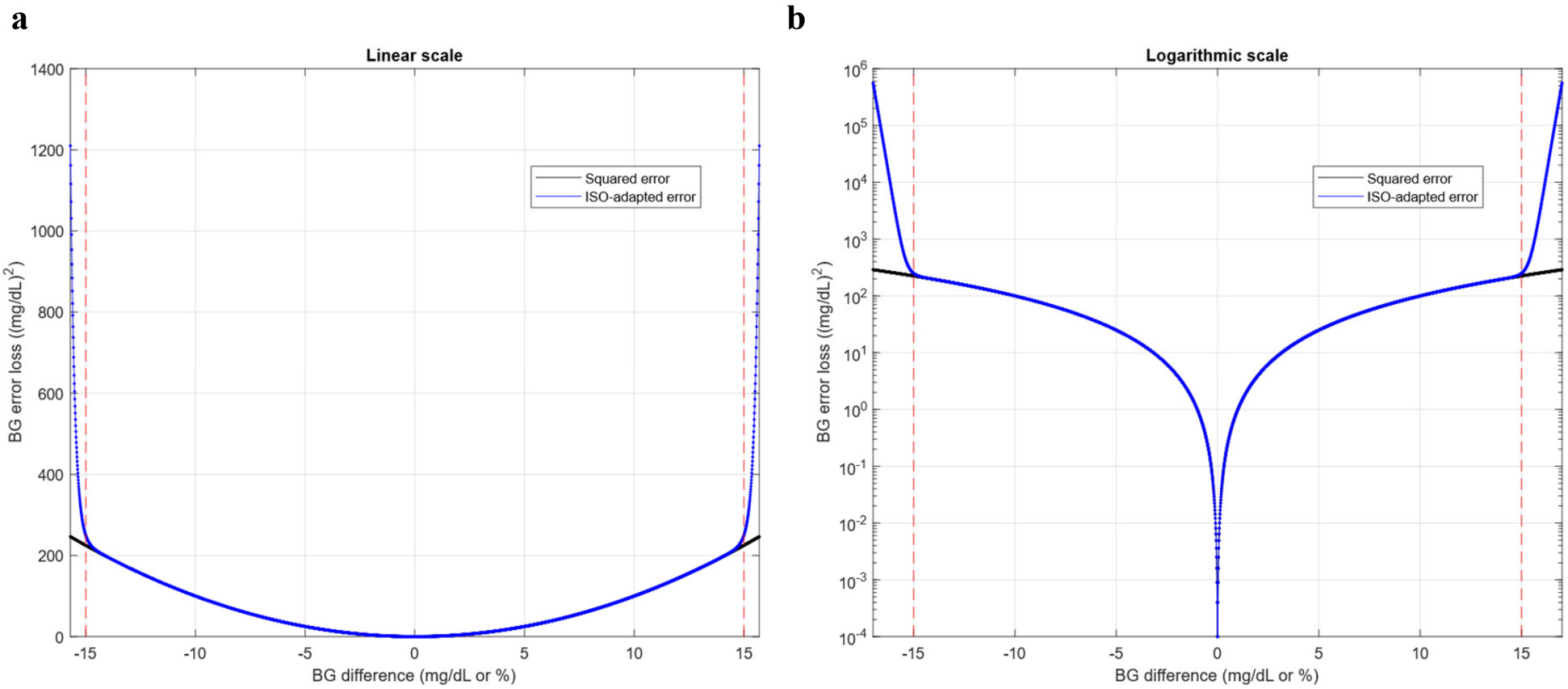
Basis of the development of the *L*_ISO_ loss function design. **(a)** Conventional squared error vs ISO 15197:2015 customized squared error in linear scale. **(b)** Conventional squared error vs ISO 15197: 2015 customized squared error in logarithmic scale. Error losses are plotted as functions of the Blood Glucose (BG) difference, i.e., the deviation of the test (predicted) glucose concentration from the reference (ground truth) value, expressed in mg/dl or percentage of the reference value according to the ISO standard. Similar behavior of both error functions is observed in the admissible range, delimited by red dashed lines.

Figure 2 highlights the different behavior of $L_{ISO}$ with respect to the squared error around the 15 mg/dl limit prescribed in ISO 15197:2015. Its steeper slope enhances the reduction of the error for non-compliant samples compared to those already within the acceptable range.

This function should be considered as a modified version of the quadratic error of a sample and therefore allows composing loss functions for collections of samples by addition or averaging as well as the use of its square root to work in the same units in which the error is defined (mg/dl in the case of interstitial glucose concentration prediction). In this work, *n* has been set to 40, and *K* set to 0.1/14^80^.

### 2.4 Proposed AI-based framework

Figure 1 shows the proposed framework to develop a personalized AI-based DIY module for interstitial glucose forecasting for people with T1D. It has been developed in Python 3.10.13 (33), using TensorFlow 2.10.0 (34) for the development of the DL models. This work was developed using an AMD Ryzen 5 3600 6-core processor and an NVIDIA GeForce RTX 4070 Ti GPU to speed up the training process of the large number of models that were generated. The choice of this approach was based on the following ideas:

a. the robustness of the DL models in the training and validation phase, considering different groups of three months within a year to train and validate the models.
b. the adaptation of the forecasting problem to the CGM case [e.g., adapting the loss function used to train the DL models based on the ISO 15197:2015 (32)].
c. the development of a fully personalized AI-based DIY module to predict interstitial glucose, preserving individuals' privacy and considering the daily habits of self-management of T1D.
d. the enhancement of the usability of this AI-based module (17) to either easily integrate it in a broader application environment and workflow or use it as a standalone tool.

Normally, each glucose sensor can be read with a dedicated reader or an application in the user's mobile phone, provided by the manufacturer. If interested, users can also have access to their raw data, which they can download. In a DIY approach, it is assumed that the users will download and deal with their data. Hence, this framework automatically reads the raw CSV data provided by the endocrinologist (in the final application would be the user itself who would upload his/her data) and prepares them to be further processed without the need for more interaction by the user.

### 2.5 Deep learning architectures

For the interstitial glucose forecasting, a *seq-to-seq* approach (35) was proposed due to its proven effectiveness in time series forecasting (36). In the *seq-to-seq* approach, the input of the model is one sequence (or more) of a certain length, and the model outputs a sequence of a certain length (usually shorter than the input for time series forecasting) that represents the subsequent time instants in the nearest future.

As a first approximation, the input sequence length was set as to 96 samples [i.e., 24 h at a sampling period of 15 min, which corresponds to an average period that includes basal glucose regulation mechanisms (37)]. This was established considering that all CGM sensors included in this study had a sampling period of 15 min, based on clinical knowledge, subject's autocorrelation studies, and considering the trade-off between the input length and the number of generated training instances. As a second input, the first difference of the CGM input window was computed to feed the DL models with additional information about CGM variations. Besides, two different PHs, have been assessed: 30- and 60-min PHs. Having a sampling period of 15 min implies an output sequence of two and four samples, respectively.

The proposed DL models were based on CNNs (38), and LSTMs (39), due to their promising results reported in time series forecasting, and specifically, in glucose levels forecasting (24–26, 40). Three different DL models, together with a baseline model, have been evaluated in this work:

1. *Naïve*: For the sake of comparison, a naïve forecasting (24) approach was included in the study. It consists of making the prediction by outputting the last values of the input sequence. This elementary prediction is considered the baseline to compare the performance of the proposed DL-based models.
2. *LSTM*: A single LSTM cell with *N* memory units, *N* being the input sequence length (set to 96 samples), followed by a fully connected layer, whose output dimension is equal to the number of predicted points.
3. *Stacked-LSTM*: A Stacked-LSTM (i.e., a model that comprises several LSTM cells) (36) composed by five layers in an encoder scheme of *N, N/2, N/2,N/4* and *N/4* memory units, from the outer to the inner layer respectively. The intention underlying this architecture is to learn the longer-term features in the layers closer to the input, and the more local temporal dependencies at the layers closer to the output. A drop-out (41) of 0.05 was added to contemplate the randomness of the studied phenomena and to avoid overfitting on the training set (42). The LSTM cells are followed by a fully connected layer whose output size is equal to the number of predicted points.
4. *Dil-UNet*: Inspired by the promising results of the U-Net one-dimensional heart sound segmentation (43), and in the successful use of CNN with dilated convolutions (25), a regression model based on a dilated U-Net has been proposed. It consists of a four-stage encoding-decoding network, with the hyperparameters outlined in Supplementary Table S1. The number of filters of the first layer was set to 4 times the number of input features. They were doubled in each encoding stage of the network and halved in its decoding part. Besides, after the last encoding convolutional layer, there are two intermediate layers that double its number of filters. There are skip connections between each encoding-decoding peer to enable direct transfer of information. The dilation rate of the convolution was added to augment the receptive field of the operations and was set to one. As with the Stacked-LSTM, several drop-out layers were added, increasing the rate to 0.1 due to the greater number of weights of this model. The last convolutional layer was connected to a fully connected layer whose output dimension is equal to the number of predicted points.

Supplementary Table S1 shows the model and training hyperparameters chosen for each architecture, which have been heuristically chosen after several trial-and-error experiments with subjects that were considered to represent the best, worst, and average subjects in terms of prediction performance. It also shows the number of parameters of each model. Notice that, due to the variation of the dense layer size that outputs the prediction sequence in the different PHs, there is a slight variation in this number for each PH. Due to the time constraints associated with the large number of independent models (one for each subject) generated in this study, hyperparameter optimization was not feasible. Notice that the training hyperparameters were the same for all architectures, seeking a fair prediction performance comparison. The batch size was set to one to compensate for the low number of samples of some of the included subjects.

Adam optimizer was used to train the models, using the standard Mean Squared Error (MSE) as the loss function, as well as using the novel $L_{ISO}$ loss previously described. Due to the extensive computational time associated with the large number of generated models in this experiment, early stopping was implemented after two epochs without reducing the loss function. The delta, (i.e., the minimum change required in the loss function value to consider a model improvement) was set to 0.0001 for both loss functions aiming at a fair comparative. This delta was considered the optimum after heuristic experimentation. In the same way, it was noticed that the models performed better when not only the CGM was considered as input, but also the CGM's first difference. Experiments also showed that introducing the second difference worsened the prediction performance. Hence, the DL models had two input features: the CGM and its first difference.

Finally, considering that hypoglycemic events are a severe health threat, the output sequences that contained samples in the hypoglycemic range were weighted the most to train the DL models, followed by the output sequences containing samples in the hyperglycemic range. Sequences that were within the target CGM range were not weighted beyond considering its occurrence including its probability in the equation. Besides, the occurrence of hypoglycemic samples is lower than the hyper- and normal CGM ranges. This together with the fact that such occurrence varies among subjects, the abovementioned weighted factor was multiplied by the probability of a given sample to be in range, or in the hyper- and hypo- ranges. This introduces the models' personalization from the training process. Depending on the number of samples in the different ranges, the weighting varies. Generally, the samples to train the models were weighted as described in Equations 4–6 for output sequences that contained hypo-, hyperglycemic samples, and samples that were in the target range, respectively. The weights multiplying the inverse of the probability of occurrence were obtained heuristically.

(4) $$W_{hypo}=2\;x\;1/p(hypo)$$

(5) $$W_{hyper}=1.1\;x\;1/p(hyper)$$

(6) $$W_{in\;range}=1/p(in\;range)$$

Where *W* represents the weights for a given subject's hypoglycemic ($W_{hypo}$), hyperglycaemic $(W_{hyper}$) and in-range ($W_{in\;range}$) sample, and *p* refers to the probabilities of a given sample to belong to the hypoglycemic range p(hypo), hyperglycaemic range p(hyper), and an in-range sample p(inrange). Notice that, for each subject, the probability is computed as the number of samples in each range divided by the total number of samples.

### 2.6 Trimester-wise 4-fold cross-validation

From the set of sequences collected, the data partition was conducted as depicted in Figure 1C. To analyze if the performance of the different AI models depends on the period of the year used to train them, which can be related to sensor misfunction, holidays period, etc., the models were cross-validated using a 4-fold approach, where each fold contained data from a consecutive 3-month period. Hence, a 9-month period and a 3-month period were used for training and validation, respectively, for each fold. For every subject, the oldest timestamp marked the beginning of the first fold. The second, third, and fourth folds were generated adding 3, 6, and 9 months to the first timestamp, respectively. Notice that there were some instances that contained data from different days, and subsequently, from different months. For the sake of consistency, borderline instances that contained data from different folds were discarded for cross-validation. Furthermore, due to the heterogeneity in the sensor reading interruptions, there were folds with more instances than others. This could have an impact on the final regression performance of the models between folds from the same subject. Finally, every fold was shuffled before feeding the DL models.

Notice that every architecture was trained and validated with the standard MSE and the $L_{ISO}$ loss functions, and this was performed for the 4 folds. Moreover, the same methodology was applied for two PHs: 30 and 60 min. Hence, and taking into account that the naïve approach does not need to be trained, for each subject, 48 AI models (3 architectures × 2 loss functions × 4 folds × 2 PHs) were generated. Considering the 29 subjects, 1,392 personalized AI-based interstitial glucose forecasting models were generated and evaluated in this work. This large number of models strongly limited the number of experiments that could be performed following this approach.

### 2.7 Prediction evaluation metrics

Commonly, prediction models for interstitial glucose level forecasting are evaluated using classical regression evaluation metrics, such as Root Mean Squared Error (RMSE), Mean Absolute Error (MAE) or Mean Absolute Percentage Error (MAPE), described in Equations 7–9, respectively, where ${\hat{y}}_{i}$ is the forecasted value, $y_{i}$ the corresponding ground truth, and *N* the number of considered values. In this work, they have been also used. However, only a few studies in the literature report the results achieved using the CEG and, to the best of our knowledge, none of them uses the complete ISO 15197:2015 (32) criteria for such evaluation since it is a highly restricted evaluation metric.

ISO 15197 is an international standard on *in vitro* diagnostic test that addresses self-testing blood glucose monitoring systems for managing diabetes mellitus within the realm of *in vitro* diagnostic test systems. These systems are designed for self-measurement by individuals without specialized medical training for the purpose of managing their condition. This standard, which has been adapted from the sensor accuracy measurement to the error between the prediction and the actual value in this work, as mentioned before in the text, establishes that the blood glucose monitoring systems (in this work, extrapolated to prediction error), must meet these two criteria:

• 95% of the measured (predicted) glucose values must be within ±15 mg/dl for blood glucose concentrations below 100 mg/dl. For values equal or greater than 100 mg/dl, the margin of error is percentage and is set at ±15%. This is illustrated in Supplementary Figure S8a.
• 99% of the measured (predicted) glucose values should fall within zones A and B of the CEG for T1D. As shown in Supplementary Figure S8b, the CEG is divided into 5 zones according to the estimated risk to the people with T1D if an outcome fails, starting with zone A, where there is no effect on clinical action, to zone E, where clinical action is altered with dangerous consequences.

Unlike the classic regression metrics, these have clinical meaning and penalize more the errors in clinically critical intervals (i.e., errors that imply misprediction of hypo- or hyperglycemia events), so they have been taken as a reference instead of the RMSE, as has been usually done (24–26, 44, 45). Hence, in this work, two additional metrics that considered these two criteria were studied: the percentage of the total predicted points within the Parkes acceptable zone (*ParkesAB*), and within the ISO acceptable zone (*ISOZone*).

(7) $$RMSE=\sqrt{\frac{1}{N}\overset{N}{\underset{i=1}{\sum }}{\left({\hat{y}}_{i}-\;y_{i}\right)}^{2}}$$

(8) $$MAE=\;\frac{1}{N}\overset{N}{\underset{i=1}{\sum }}|{\hat{y}}_{i}-\;y_{i}|\;$$

(9) $$MAPE=\;\frac{1}{N}\overset{N}{\underset{i=1}{\sum }}\frac{\left|{\hat{y}}_{i}-\;y_{i}\right|}{y_{i}}*100\;\text{\%}$$

### 2.8 Do-it-yourself module design and containerization

After performing the personalized AI-models development and validation, a Python application was developed to encapsulate the DIY framework in a usable module. Additionally, the Docker tool (46, 47) was used to containerize the app, enabling its use in different Operating Systems (OSs), including those used in smartphones. In this work, this module was validated in Windows 10 and Ubuntu LTS 24.04, to prove that this module was OS-agnostic. Only the LSTM for 30-min PH was implemented in this stage, since it was the model with the shortest training times, and this was sufficient to test the DIY module functionality.

The design of the application aimed to increase the users' empowerment (30) regarding their data and the AI models generation using them. Considering the inclusion criteria, the preprocessing and the obtained results regarding prediction performance of this work, there are cases where the implementation of this AI-based framework is not feasible, e.g., to provide less than a year of data to generate the model, the fact that such year of data does not provide enough samples to properly train the DL architectures, etc. All this information will be prompted to the users when needed. The users will also have the option to skip this information in case they are not interested. Four main scenarios were considered for the design of this tool. A key point is that all these scenarios are triggered by the user by calling the application. In the current stage, this means uploading their CGM sensor data to the DIY module. The possible scenarios that the tool contemplate are:

1. User's first use providing enough data. The DL models are generated and training following a 4-fold cross-validation approach. The best model is selected and locally stored to be the one that will provide the personalized CGM predictions.
2. User's first use without providing enough data. No DL model will be generated. The application will encourage the user to upload her/his again, indicating why user's data was not suitable for the CGM prediction (if desired).
3. User's second (or beyond) use providing enough and uninterrupted data (i.e., one day with the proposed approach, as it is the input length of the DL models). The model generated in 1) is called and the CGM prediction is provided.
4. User's second (or beyond) use and not providing enough data. No prediction will be provided, and, if the user wants so, an explanation about why the prediction did not take place will be provided.

The design of the module interface, including the graphics, was developed through a co-creation process. Potential users participated in focus groups, where they expressed their needs and preferences related to the module. One of the objectives of these sessions was to find the best way to represent the glucose data of the potential users. To achieve this, they were asked about their preferences and were shown examples of graphs. Participants chose the ones they found most useful for decision-making regarding their insulin dosage. A total of six focus groups were conducted with participants with T1D (*n* = 31).

## 3 Results

### 3.1 Validation of the proposed ISO-adapted loss functions for DL model training

**Figure 3:**
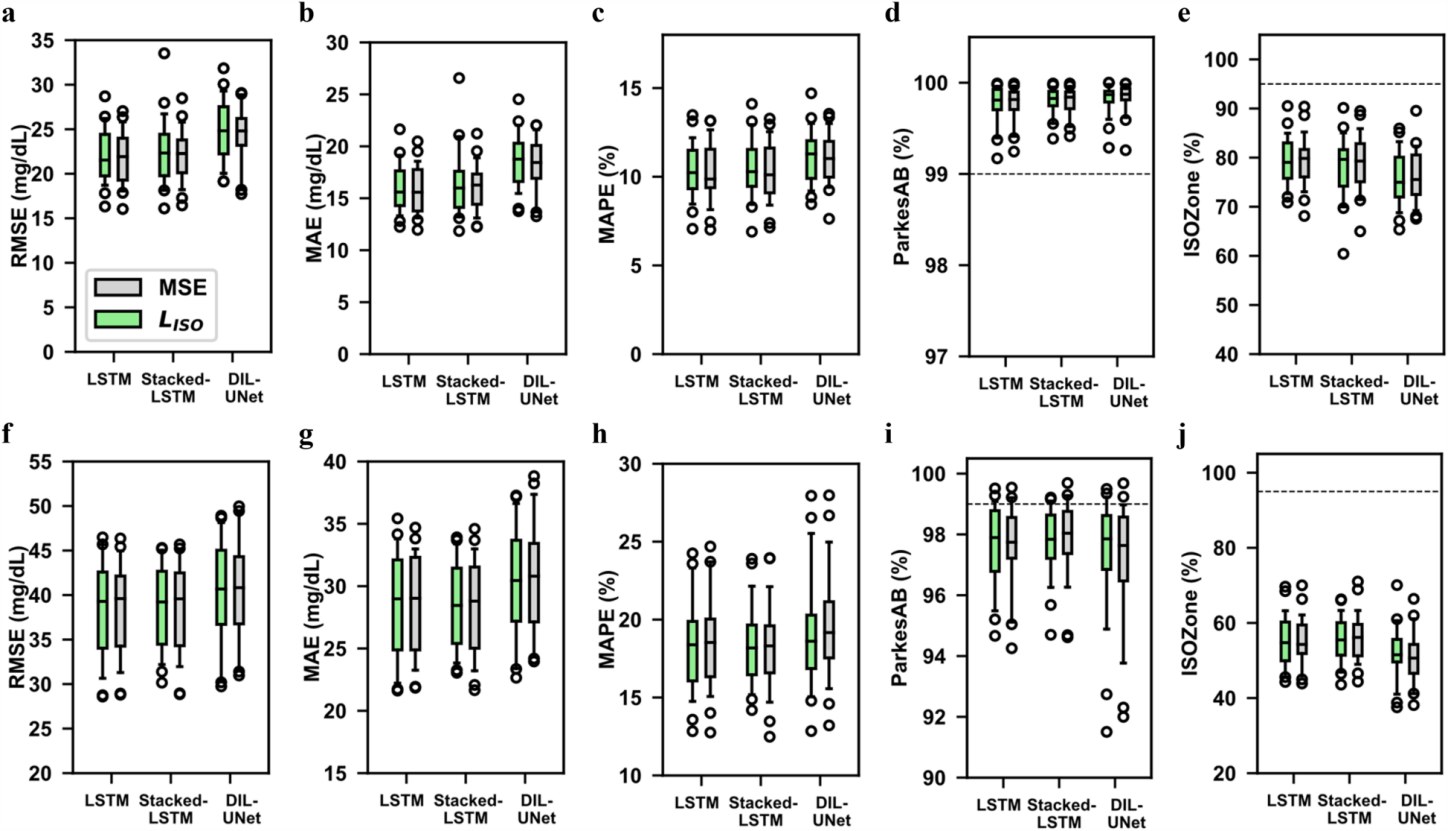
Comparison of metric performance for the proposed loss functions: MSE and *L*_ISO_. Boxplots of the evaluation metrics in all subjects’ (*n* = 29) four validation folds in the three proposed DL models training them with the standard MSE (grey) and the *L*_ISO_ function (green) for 30- (top) and 60-min (bottom) predictions. In the boxplots, centre line represents the median, box comprises second and third quartiles, whiskers extend up to 1.5 times the interquartile range, and circles denotes outliers. The naïve model was not included because it does not require any training. The lower the RMSE, MAE and MAPE, the better the prediction performance. The higher the ISOZone and ParkesAB, the better the prediction performance. The dash lines represent the ISOZone and ParkesAB minimum requirements according to the ISO 15197:2015 standard. **(a)** RMSE, 30-min PH. **(b)** MAE, 30-min PH. **(c)** MAPE, 30-min PH. **(d)** ParkesAB, 30-min PH. **(e)** ISOZone, 30-min PH. **(f)** RMSE, 60-min PH. **(g)** MAE, 60-min PH. **(h)** MAPE, 60-min PH. **(i)** ParkesAB, 60-min PH. **(j)** ISOZone, 60-min PH.

Figure 3 shows the comparison of the prediction performance when training the LSTM, Stacked-LSTM and Dil-UNet models with $L_{ISO}$ (green) and the standard MSE (grey). Notice that the naïve model does not require any training and hence any loss function. The upper part of Figure 3 illustrates the metrics for 30-min PH, and the lower part for 60-min PH. This evaluation was performed in terms of RMSE, (Figures 3a,f), MAE, (Figures 3b,g), MAPE, (Figures 3c,h), *ParkesAB*, (Figures 3d,i), and *ISOZone*, (Figures 3e,j). They comprise the results obtained for the included 29 subjects. Notice that for RMSE, MAE, and MAPE, the lower their value, the better the prediction performance, zero being the ideal value. Conversely, *ParkesAB* and *ISOZone* are percentage metrics, where the ideal case would be to have 100% of the points within the acceptable zones. Nonetheless, a *ParkesAB* value of 99% and an *ISOZone* value of 95% are compliant with the ISO 15197:2015 standard (32).

These results show that the proposed $L_{ISO}$ training provided a similar prediction performance than the standard MSE for all models and for both PHs. Both $L_{ISO}$ and MSE showed higher variability for the 60-min PH (Figures 3f–j), than for the 30-min PH (Figures 3a–e). The 30-min prediction implies less uncertainty than the 60-min prediction and, therefore, both error magnitude and variability increase in the latter case. MSE and $L_{ISO}$ training presented equivalent performance, although the median metrics in MSE metrics showed slightly better performance than $L_{ISO}$. In the case of the 60-min prediction, $L_{ISO}$ training improves the Dil-UNet performance in terms of all metrics (Figures 3f–j). Besides, this improvement is the largest among all the models comparing both loss functions. Considering that the 60-min PH is a more complex task than the 30-min analogue, and that the Dil-UNet has around 15 and 8 times more parameters than LSTM and Stacked-LSTM respectively (see Supplementary Table S1), this suggests that the larger the PH and the more complex the model, the more evident is the $L_{ISO}$ influence in glucose forecasting. Besides, it can also be observed that the performance limitations inherent to the proposed DL architectures are not overcome by the use of any loss function. As an example, the use of $L_{ISO}$ to train the Dil-UNet, improves the prediction performance compared to the MSE training, but it does not outperform the Stacked-LSTM or the LSTM.

### 3.2 Comparison between different models for personalized interstitial glucose forecasting

Tables 1, 2 show the mean and Standard Deviation (SD) of the obtained results for 30-min and 60-min PHs, respectively, considering the four validation folds in all subjects (*n* = 29) for the four evaluated models. Results are grouped by the loss function used to train the DL models.

**Table 1:** Evaluation metrics in the validation folds for the proposed models for MSE and $L_{ISO}$ loss functions and the 30-min PH. Results are shown as mean ± SD of the included subjects (*n* = 29). Results in bold font indicate the model with the best performance for a specific metric.

**Loss function = MSE**					
**Metric**	**RMSE (mg/dl)**	**MAE (mg/dl)**	**MAPE (%)**	***ParkesAB* (%)**	***ISOZone* (%)**
**Model**					
**Naïve** ^a^	26.74 ± 3.86	19.50 ± 3.00	12.63 ± 2.02	99.41 ± 0.48	71.91 ± 5.67
**LSTM**	**21.86** ± **2.87**	**15.83** ± **2.21**	**10.28** ± **1.57**	99.77 ± 0.18	**79.34** ± **4.97**
**Stacked-LSTM**	21.98 ± 2.86	15.95 ± 2.18	10.34 ± 1.64	99.80 ± 0.15	78.91 ± 5.81
**Dil-UNet**	24.46 ± 3.14	18.28 ± 2.42	11.08 ± 1.38	**99.84** ± **0.15**	76.25 ± 5.03
**Loss function =** $L_{ISO}$					
**Metric**	**RMSE (mg/dl)**	**MAE (mg/dl)**	**MAPE (%)**	***ParkesAB* (%)**	***ISOZone* (%)**
**Model**					
**Naïve** ^a^	26.74 ± 3.86	19.50 ± 3.00	12.63 ± 2.02	99.41 ± 0.48	71.91 ± 5.67
**LSTM**	**22.05** ± **2.94**	**16.00** ± **2.22**	**10.35** ± **1.53**	99.76 ± 0.20	**79.20** ± **4.77**
**Stacked-LSTM**	22.39 ± 3.91	16.34 ± 3.04	10.49 ± 1.61	99.80 ± 0.13	78.11 ± 6.13
**DIL-UNet**	24.84 ± 3.30	18.60 ± 2.58	11.23 ± 1.49	**99.83** ± **0.15**	75.57 ± 5.40

^a^ Naïve model does not require any training, but its metrics are placed to compare this baseline model to the rest of the proposed models.

**Table 2:** Evaluation metrics in the validation folds for the proposed models for MSE and $L_{ISO}$ loss functions and the 60-min PH. Results are shown as mean ± SD of the included subjects (*n* = 29). Results in bold font indicate the model with the best performance for a specific metric.

Loss function = MSE					
Metric	**RMSE (mg/dl)**	**MAE (mg/dl)**	**MAPE (%)**	***ParkesAB* (%)**	***ISOZone* (%)**
Model					
**Naïve** **^a^**	44.64 ± 6.78	33.18 ± 5.31	21.75 ± 3.66	96.17 ± 2.03	50.61 ± 6.01
**LSTM**	38.50 ± 5.13	28.58 ± 3.96	18.39 ± 2.88	97.68 ± 1.36	55.29 ± 6.35
**Stacked-LSTM**	**38.38** ± **4.84**	**28.33** ± **3.59**	**18.15** ± **2.69**	**97.79** ± **1.23**	**56.09** ± **6.11**
**Dil-UNet**	40.25 ± 5.71	30.63 ± 4.31	19.70 ± 3.47	97.13 ± 2.00	51.06 ± 6.82
Loss Function = $L_{ISO}$					
**Metric**	**RMSE (mg/dl)**	**MAE (mg/dl)**	**MAPE (%)**	***ParkesAB* (%)**	***ISOZone***
**Model**					
**Naïve** ^a^	44.64 ± 6.78	33.18 ± 5.31	21.75 ± 3.66	96.17 ± 2.03	50.61 ± 6.01
**LSTM**	38.52 ± 5.18	28.60 ± 3.92	18.44 ± 2.90	97.67 ± 1.31	55.19 ± 6.56
**Stacked-LSTM**	**38.44** ± **4.51**	**28.44** ± **3.29**	**18.33** ± **2.52**	**97.77** ± **1.07**	**55.65** ± **5.93**
**DIL-UNet**	40.00 ± 5.66	30.31 ± 4.30	19.27 ± 3.57	97.43 ± 1.98	51.83 ± 6.97

^a^ Naïve model does not require any training, but its metrics are placed to compare this baseline model to the rest of the proposed models.

The three proposed DL models outperformed the naïve approach (set as baseline for the minimum requirements regarding prediction performance) for all metrics and both PHs. These results show that, among the proposed DL models, there is no architecture that works optimally for both PHs. Moreover, LSTM-based models outperform the Dil-UNet for both loss functions and PHs. This might be related to the fact that a recurrent neural network as the LSTM and Stacked-LSTM are based on an architecture that implements memory cells that preserve their state over time, theoretically more suitable for time series prediction purposes than other architectures that are in principle more complex but are not specifically designed to catch the temporal dependencies (48). Besides, regardless of the employed loss function to train the models, LSTM showed slightly better prediction metrics for 30-min PH, whereas Stacked-LSTM, which has more parameters (see Supplementary Table S1), did it for 60-min PH.

LSTM and Stacked-LSTM presented slightly better results when trained with the standard MSE than when trained with $L_{ISO}$. Focusing on the difference of all metrics, it is noticeable that $L_{ISO}$ influences more the Stacked-LSTM than the LSTM. This could be related to the fact that the Stacked-LSTM possesses twice the number of parameters than LSTM (see Supplementary Table S1) and hence is more “flexible” than the LSTM.

Finally, focusing on the diabetes-specific metrics, it is worth noticing that the *ParkesAB* criterion (i.e., to be greater than 99%) was fulfilled by all models regardless of the loss function used for the 30-min PH. However, this requirement was not fulfilled for the 60-min PH. All models left from 2.3% to 3% below the minimum threshold. The *ISOZone* metric, which is the most restrictive requirement based on the ISO 15197:2015 standard (32), was not fulfilled in any case (i.e., to be greater than 95%), for any of the PHs, being around 16% and 39% below the threshold for 30-min and 60-min PHs, respectively. This might be related to the architectural limitations of the proposed DL models.

### 3.3 Assessing inter- and intra-subject variability through a subject-wise evaluation

**Figure 4:**
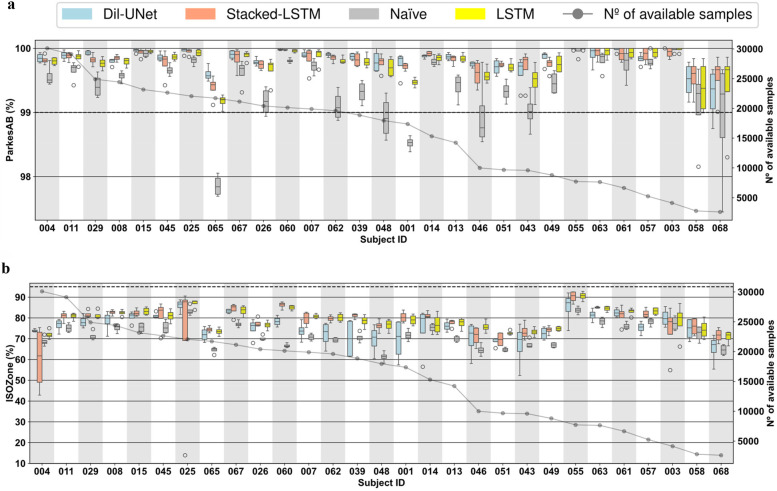
Boxplots representing subject-wise (*n* = 29) ISO-based prediction metrics (parkesAB and ISOZone) computed with the four validation folds (PH = 30 min) for the naïve model, LTSM, stacked-LSTM, and Dil-uNet after being trained with *L*_ISO_. The subjects are sorted in descending order of available instances to train the models. In the boxplots, the centre line, the box limits and the whiskers represent the median, the upper and lower quartile and the 1.5× interquartile range, respectively. **(a)** ParkesAB. **(b)** ISOZone.

**Figure 5:**
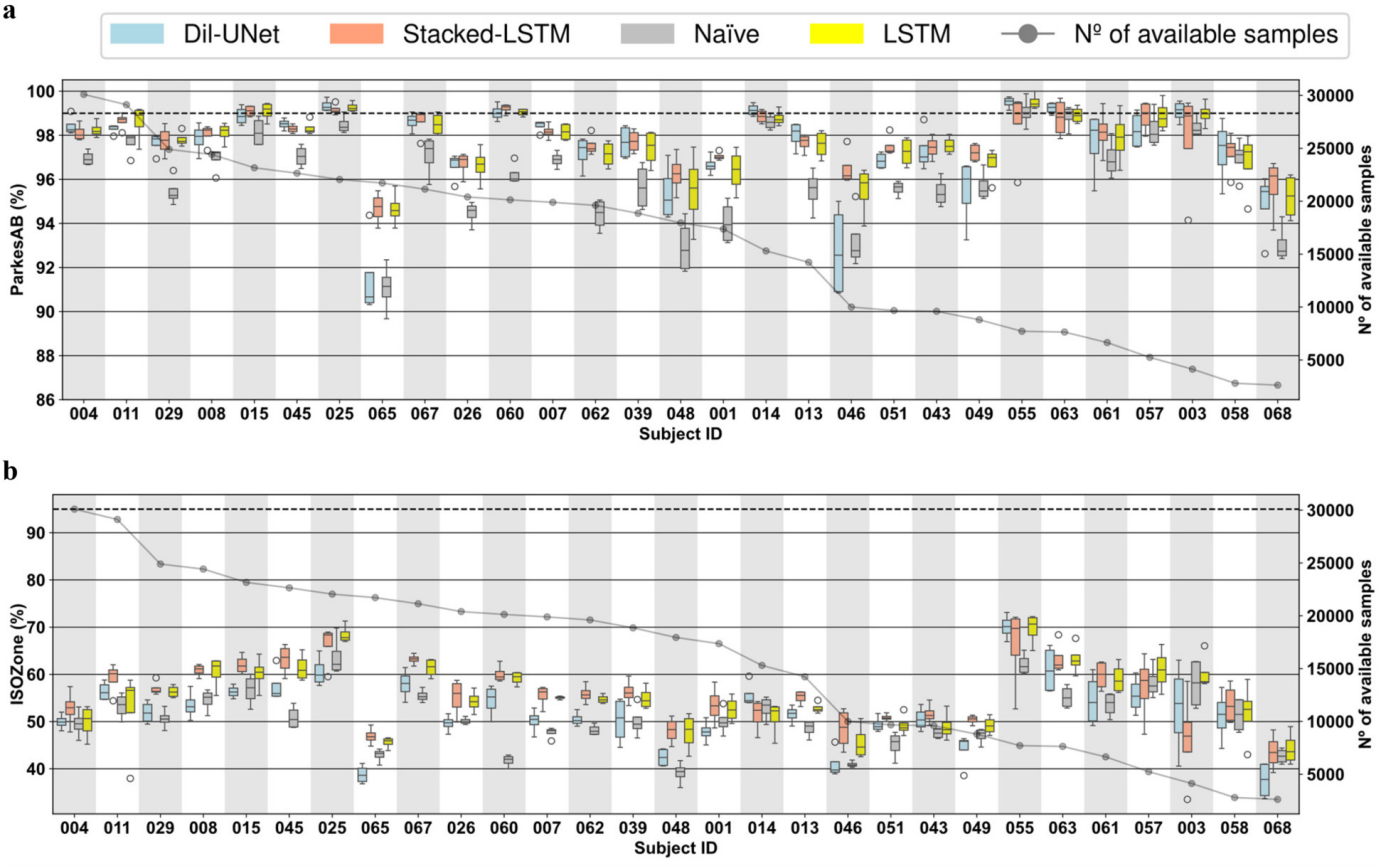
Boxplots representing subject-wise (*n* = 29) ISO-based prediction metrics (parkesAB and ISOZone) computed with the four validation folds (PH = 60 min) for the naïve model, LTSM, stacked-LSTM, and Dil-uNet after being trained with *L*_ISO_. The subjects are sorted in descending order of available instances to train the models. In the boxplots, the centre line, the box limits and the whiskers represent the median, the upper and lower quartile and the 1.5× interquartile range, respectively. **(a)** ParkesAB. **(b)** ISOZone.

The results presented in the previous section provided insights about the overall differences between the DL models evaluated for interstitial glucose forecasting but did not consider the inter- and intra-subject variability (49, 50). Due to the fact that this work presents a DL-based DIY module that follows a subject-oriented approach, an individual evaluation of the DL models that would derive in personalized AI feedback is essential. Figures 4, 5 shows the subject-wise diabetes-specific prediction metrics, namely *ParkesAB* and *ISOZone* metrics after training the models only with $L_{ISO}$ loss functions for 30-min and 60-min PHs, respectively. Supplementary Figure S2, Supplementary Figure S3 show the *ParkesAB* and *ISOZone* metrics after training with the MSE for 30- and 60-min PH, respectively, which are equivalent to those obtained after the $L_{ISO}$ training. Subjects' IDs were sorted by the number of available CGM samples to train and validate the DL models following the proposed 4-fold trimester-wise cross-validation approach. These two metrics have been analysed in detail because they contemplate the prediction errors that are clinically relevant and are specific for the interstitial glucose prediction task. Additionally, RMSE was also analysed for 30- and 60-min PH after training with both loss functions (Supplementary Figure S4, Supplementary Figure S5, respectively), since it is the metric that is usually taken as the reference to compare between different models in the literature.

From Tables 1, 2, no significant differences are observed between the means in any of the evaluated metrics. In all cases, such differences are clearly lower than the average SD. This implies that there are not statistically significant differences in favor of any of the metrics.

It is worth noting that, although a sufficient number of samples is required to properly train a DL model, it can be observed that more CGM samples do not imply more accurate predictions in the proposed DL models. This effect is related to: (1) the glucose patterns heterogeneity among subjects (i.e., inter-subject variability) (49), specially in people with T1D (50); (2) the day-to-day CGM variability (i.e., intra-subject variability) (51); and (3) the various factors that influence the subject's glucose dynamics (such as insulin administration, subject metabolism, carbohydrate intake, physical activity) that are not contemplated in the proposed DL models training because only CGM data were employed in this study.

In concordance with the results exposed in the global validation (Table 1), in the vast majority of the cases the LSTM-based models presented better prediction performance in terms of RMSE (Supplementary Figure S4, Supplementary Figure S5) than the naïve model for both PHs, even though for some subjects the naïve model showed lower variability (e.g., subjects #045, #048, and #068, Supplementary Figure S4a). In the case of Dil-UNet, most of the cases presented lower median than the naïve model but its strong dependency on the fold (i.e., high intra-subject variability) makes it a less robust model than the naïve approach for a few subjects (e.g., subject #48, Supplementary Figure S4b). Dil-UNet showed the largest variability within the four validation folds for both PHs (Supplementary Figure S4, Supplementary Figure S5) in almost all subjects trained with both loss functions, with a few exceptions (e.g., subject #004 and #014, Supplementary Figure S4a). This means that, in general and for both PHs, the Dil-UNet prediction performance is more dependent on the folds (i.e., more sensible to the intra-subject variability) than the LSTM-based models. Thus, the interstitial glucose prediction reliability depends more on the time of the year used for the model training, making it a less robust model in terms of RMSE than the LSTM-based models. This could be related to the fact that Dil-UNet has a significantly larger number of parameters than the other two models (see Supplementary Table S1), so more samples might be needed to achieve better prediction performance.

The improvement presented by the proposed models compared to the naïve model becomes more evident studying the *ParkesAB* metric for both PHs. The training of the DL models using both loss functions outperformed the naïve approach, although in a few cases some models presented slightly higher variability (e.g., subject #007, Figure 4a). In the case of 30-min PH (Figure 4a; Supplementary Figure S2a), the only exceptions were subjects #068 and #058, which are the ones that had the fewest available samples for training. In the case of 60-min PH, the three DL models of the seven subjects with fewer samples (#063, #055, #061, #057 #003, #068, and #058) presented equivalent *ParkesAB* (Figure 5a; Supplementary Figure S3a). This happened with the training of both functions, but it was more evident with $L_{ISO}$ training. Apart from these exceptions related to the lack of enough samples, LSTM-based models always showed higher *ParkesAB*, and also Dil-UNet (except for subjects #065 and #046).

Prediction performance in terms of *ParkesAB* and *ISOZone* evidences the RMSE limitations to properly characterize the interstitial glucose forecasting model performance. Firstly, model variability among folds related to the lack of training samples showed by the diabetes-specific metrics was not reflected by RMSE. Besides, *ParkesAB* and *ISOZone* give more importance to samples that are clinically relevant (i.e., prediction errors of hypoglycemic and hyperglycemic events), it therefore provides more information on whether the prediction model is suitable for this specific task. As an example, comparing RMSE and *ParkesAB* metrics for the 30-min PH training with MSE loss function (Supplementary Figure S4a, Supplementary Figure S2a, respectively) of subjects #067 and #025, it is observed that #067 presented lower RMSE (i.e., better prediction) in all models than subject #025. Nonetheless, subject #025 presented a higher *ParkesAB* (i.e., better prediction in the diabetes context). Hence, even though subject #067 predictions showed a lower RMSE, the predictions of subject #025 will be more clinically reliable and relevant. Thus, lower RMSE does not always imply better interstitial glucose predictions for people with T1D. This was consistent when comparing also the RMSE with *ISOZone* (Figures 4b,5b; Supplementary Figure S2b, Supplementary Figure S3b).

Besides, even though more training samples do not ensure better prediction performance, obtained results have set a threshold regarding available training CGM samples to achieve a fairly reliable glucose prediction. Such threshold is different for each PH. Setting the limit where the naïve approach (i.e., the predicted sequence consists of the last samples of the input) achieves equivalent *ParkesAB* and *ISOZone* in terms of median and SD than all three proposed DL models, the threshold for 30-min PH is ∼1,500 CGM training samples, whereas for 60-min PH it is ∼5,000. Such a difference might be related to the complexity of the task. A more complex prediction requires, in principle, more training samples to properly learn the phenomenon. This phenomenon occurred for both loss functions and both PHs (Figures 4c,d, 5c,d). Notice that both diabetes-specific metrics show consistency in setting these thresholds. Hence, the proposed DIY module will discard potential users that provide CGM data that would end in a training and validation dataset with less training samples than the correspondent threshold, since it has been demonstrated that this number is not enough to generate a reliable interstitial prediction model using the proposed DL models.

*ParkesAB* metric is based on the least restrictive requirement of ISO 15197:2015 (32), that establishes that at least 99% of the prediction errors must fall within certain limits considering the clinically critical glucose concentrations. A DL model whose predictions meet this requirement can be considered clinically safe under this criterion. Studying this metric for the 30-min PH (Figures 4c,d training with $L_{ISO}$, and Supplementary Figure S2a, Supplementary Figure S3a training with MSE) the three proposed DL models met this requirement within the four validation folds of the 29 subjects. Also, the naïve approach, except for subjects #065 and #001, met this ISO requirement, which explains why the most restrictive criteria included in the ISO standard has been also evaluated in this work (i.e., *ISOZone*). Conversely, for the 60-min PH, only 7 out of the 29 subjects met this criterion training with MSE (Supplementary Figure S3a), and 9 out of 29 subjects training with $L_{ISO}$ (Figure 5A). Hence, even though Table 1 shows unfavorable results of the proposed novel loss function, more subjects are included in the *ParkesAB* acceptable zone for 60-min PH. This evidences the need for performing a subject-oriented evaluation of the DL models when proposing approaches such as a DIY using diabetes-specific metrics (52). However, the results obtained reveal the need for further improvement in the proposed architectures.

*ISOZone* metric, is the most restrictive condition of ISO 15197:2015 (32), setting the minimum of the prediction errors in 95% within the acceptable zone. Thus, fulfilling this requirement would lead to more clinically safe actuations than only fulfilling *ParkesAB* requirement. Unfortunately, the *ISOZone* metric did not surpass 95% in any personalized DL model, confirming the fact further improvements in the architectures should be assessed (Figures 4b,5b; Supplementary Figure S2b, Supplementary Figure S3b).

Finally, assessing the inter-subject variability, it is observed that none of the three proposed DL models work optimally for all subjects, although LSTM-based models presented the best average prediction performance (Table 1). For example, for the 60-min PH, Dil-UNet prediction in subject #055 shows the best *ParkesAB* trained with $L_{ISO}$, slightly better than LSTM-based models. However, the same model presented by far the worst *ParkesAB* in subject #65 compared to LSTM-based models, and slightly worse than the naïve approach (Figure 5a). The same occurs with the *ISOZone* (Figure 5b). Regarding RMSE, the Stacked-LSTM model trained with MSE presented relatively low variability within folds for both PHs and loss functions, although there are some exceptions (Supplementary Figure S4, Supplementary Figure S5). However, mean and SD variations in all metrics are comparable within all models. Considering that besides the inter-subject variability, the CGM samples of the subjects can present different day-to-day and intra-subject variability (51), this suggests that the variability and prediction capabilities are strongly dependent on the subject. There is not a DL model that clearly outperforms the rest, but all of them present better prediction performance in the same subjects, although there are some variations related to how a certain architecture has learnt about a given trend of the subject's CGM data.

### 3.4 Comparison with other state-of-the-art studies

There are many studies in the scientific literature that have assessed the glucose forecasting problem. However, for a fair comparison with this work, the following criteria were used for the selection of other studies:

a. Explicit indications that the glucose prediction was performed in people with T1D.
b. Reported results of 30-min and/or 60-min PHs.
c. Reporting results from a validation and/or test set of real glucose data.
d. The number of subjects included in the evaluation, i.e., *n*, was greater than 5.

Table 3 shows the comparison between the included studies. It is worth noticing that all the subjects included in the dataset collected and used in our work had sensors with a sampling period of 15 min. This limits the temporal information provided to the DL models compared to the other considered studies that included CGM data sampled every 5 min. This could be partially mitigated by increasing number of input time steps of the DL models. Moreover, our work and the work presented by De Paoli et al. (40) were the only ones that fed the models with solely CGM data. This also justifies that the personalized models presented here had the largest input window length within the compared models.

**Table 3:** Comparison of the proposed DIY framework with other state-of-the-art approaches. Results for 30- and 60-min PHs are shown as mean ± SD (when both reported). Best results are highlighted in bold for each metric. Only test or validation results are included. (*n*, number of subjects to evaluate the models; SP, sampling period of the sensors considered in the work; *N*, input window length; Pers. Models: if personalized models were developed in the work; N/A, result not available in the original work).

Model (#subjects)	SP (min)	inputs	N (time)	Train/validation/test periods	Pers. models	30-min PH			60-min PH		
						RMSE (mg/dl)	*ParkesAB* (%)	*ISOZone* (%)	RMSE (mg/dl)	*ParkesAB* (%)	*ISOZone* (%)
Proposed LSTM and stacked-LSTM^a^ (*n* = 29)	• 15	- CGM - CGM 1st derivative	96 (24 h)	9 months/3 months/(4-fold cross-validation)	Yes	21.86 ± 2.86	**99.77** **±** **0.19**	**79.34** **±** **4.95**	38.44 ± 4.55	**97.77** **±** **1.06**	**56.09** **±** **6.13**
Multi-input dilated CNN (24) (*n* = 97)	5	- CGM - Meal intake - Basal insulin rate - Bolus insulin	Multi-step of 36 (3 h)	143.42 ± 90.39 days/-/25.37 ± 4.96 days	No	23.22 ± 6.39	N/A	N/A	N/A^b^	N/A	N/A
Multi-input dilated CNN (25) (*n* = 10.6)	5	- CGM - Insulin - Meal intake - Time stamps	Sliding window of 16 (1.25 h)	*Dataset 1*: 90 days/-/90 days *Dataset 2*: 40 days/-/10 days	No	19.19 ± 2.74 19.28 ± 2.76	N/A	N/A	31.78 ± 4.94 31.83 ± 3.49	N/A	N/A
Jump neural network (40) (*n* = 10)	–	- CGM	N/A (10 min)	1 day/-/1 day or more	Yes	24.9	N/A	N/A	N/A	N/A	N/A
LSTM (26) (*n* = 150)	5	- CGM - Insulin bolus	36 (3 h)	105 subjects/ -/45 subjects	No	19.8 ± 3.2	99.60	N/A	33.2 ± 5.4	97.60	N/A
Modified N-BEATS (44) (*_n_ *_= 6)_	5	- CGM - Finger stick glucose - Insulin bolus - Carbohydrate - Sine of time - Cosine of time	12–42 (1 h—∼3 h)	∼10,000 samples per subject/-/∼2,500 samples per subject	No	18.22	99	N/A	31.66	N/A	N/A

^a^ LSTM was the model selected for 30-min PH, and Stacked-LSTM for 60-min PH.

^b^ Evaluation was performed with a smaller subset of 24 subjects that provided more favourable prediction metrics.

Focusing on De Paoli's work, it is also the only one reporting the development of a personalized model for each subject. Hence, a fair comparison can be assessed. However, they did not report prediction performance at 60-min PH, and neither did diabetes-specific metrics. Our proposed framework outperformed the results obtained by De Paoli's work, which is based on Jump Neural Networks (40), in terms of RMSE at 30-min PH. Thus, among the only two studies that reported personalized models using only CGM data, our work provided the best prediction performance.

Although the prediction performance of the DIY-based models is mainly limited by the use of only CGM data, our models achieve comparable performance in terms of RMSE to the rest of considered works for 30-min PH. For 60-min PH, these differences are more significant. The model that presents the best RMSE for both PHs is the N-BEATS (44). For 30-min PH, our LSTM showed similar performance (21.86 ± 2.86 vs. 18.22). This difference is more noticeable for 60-min PHs, where N-BEATS outperforms the Stacked-LSTM (38.44 ± 4.55 against 31.66). Nonetheless, this work only evaluated this model with 6 subjects, whereas we used 29. Moreover, they used several variables as input, including CGM, finger stick glucose, insulin, and carbohydrates intake, among others. It is also worth noticing that our work is the only one reporting *ISOZone* metric, which is the most restrictive criteria of the ISO 15197:2015 standard. Hence, this can serve as a benchmark for comparison in future developments of processing algorithms.

Finally, the *ParkesAB* metric, which is a metric that indicates if the clinical decision based on the predicted CGM labels is clinically safe for the subject, was reported only in two of the studies found in the literature. For the 60-min PH, only one work reported this result without reaching the minimum requirement (≥99%), but our work was closer to the minimum than the LSTM-based model proposed by Mosquera-Lopez et al. (26) (97.77% and 97.60%, respectively). For the 30-min PH, the two studies that reported this metric fulfilled the *ParkesAB* requirement. It is worth noticing that the personalized DIY DL models developed in this work presented the best *ParkesAB* metric for both evaluated PHs, evidencing again the limitations of the RMSE as the standard for glucose prediction models comparison. Thus, this approach can be considered the clinically safest among the ones included in this comparative, being the only one that followed a subject-oriented approach for the DL training and validation using only 1-year CGM data from one subject to train the model. Unfortunately, the evaluation of the proposed framework using the OhioT1DM dataset (the most common dataset for glucose forecasting benchmarking) (53) was not feasible. Our work uses trimester-wise partitions based on 1 year of CGM data, and the OhioT1DM dataset provides only 2 weeks of diabetes-related readings, making impossible to do a fair comparison.

### 3.5 Evaluation of the DL models using a test set

**Figure 6:**
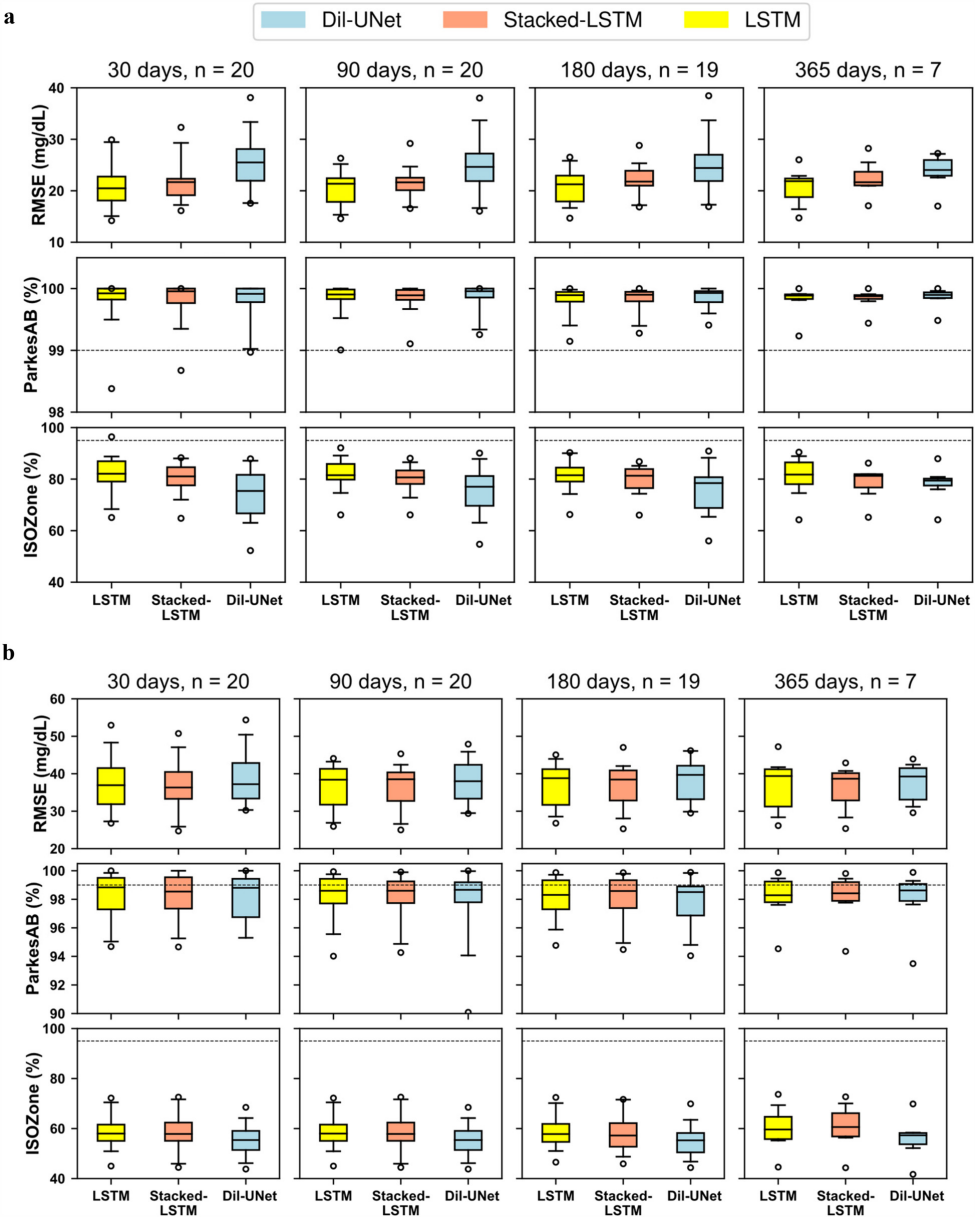
RMSE, parkesAB, and ISOZone metrics for different test sets after training the personalized models using 1 year of CGM data. The test sets included the first 30, 90, 180, 365 days for each subject (when available). The number of subjects with available data for each experiment is indicated with *n* evaluated with different number of days. For each particular test set the number of subjects with enough available data is indicated with n. The dash line represents the minimum percentage of points in ParkesAB and ISOZone to fulfil the ISO standard. In the boxplots, centre line represents the median, box comprises second and third quartiles, whiskers extend up to 1.5 times the interquartile range, and circles denote outliers. **(a)** 30-min PH. **(b)** 60-min PH.

As a final validation of the DIY DL-based framework for personalized interstitial glucose prediction, additional data from the 29 subjects included in this work were collected to further evaluate the consistency of the above presented results. For every subject, a DL-model was generated using the whole year that was previously partitioned following a 4-folds using $L_{ISO}.$ Then, the new collected test set was used to evaluate the models. Sensor replacement or data unavailability due to reading interruptions produced a decrease in the number of subjects available for this evaluation. The number of days included in the test set was varied, including 30, 90, 180, and 365 days. This was assessed to analyze if the prediction performance differed depending on the length of the test set. As shown in Figure 6, from the initial 29 subjects, three test sets of subjects were obtained: (1) 20 subjects for testing 30 and 90 days; (2) 19 subjects for testing 180 days; and (3) 7 subjects for testing 365 days. RMSE, *ParkesAB,* and *ISOZone* metrics were analyzed. The boxplots comprise data from *n* subjects, being *n* dependent on the number of days of the test set. Moreover, Supplementary Tables S3, Supplementary Table S4 show the mean and SD corresponding to these results for 30- and 60-min PH, respectively.

Figure 6; Supplementary Tables S3, Supplementary Table S4 show that, for both PHs, the prediction performances barely change when increasing the number of days included in the test set. The greatest change in terms of the median and variability is observed when the test set includes 365 days. This is more evident for 60-min PH (Figure 6B). However, this is likely related to the significant decrease in the number of subjects included. It can be concluded that, following the DIY-approach after training with 1 year of CGM data, testing with one month of data is representative of the overall personalized model performance.

Comparing results of Table 1, that shows the prediction metrics after training the models with 9 months, to Supplementary Tables S3, Supplementary Table S4, that shows RMSE, *ParkesAB* and *ISOZone* metrics after training with the whole year, it is observed a general improvement in RMSE and diabetes-specific metrics. However, the 4-folds cross-validation included 29 subjects, and in the test only 20 subjects were included (for 30 and 90 testing days) due to data unavailability. Thus, this performance improvement might be related both due to training with more data (∼25% more, assuming equal distribution of the CGM readings within the year), and due to include less subjects in the evaluation. Furthermore, it is worth noting that for a 60-min PH, the difference between the Dil-U-Net and the LSTM-based models are less, suggesting that the former benefits more from being trained with more data than the LSTM-based. Concluding, the use of an additional test set validates the prediction performance previously shown and, hence, the competitive performance of the DIY model proposed in this work respect the state-of-the-art.

### 3.6 DIY module implementation and validation in different operating systems

The design of this open-source module has considered its portability for its standalone use, its easy integration in broader applications regardless of the OS running the software, and its modular design so that researchers and developers can test novel architectures following the proposed DIY approach. To prove that this module is OS-agnostic (i.e., it can run over different OSs), the Python application has been deployed to run in a Docker (46) container, proving its functionality in two different OSs: Windows 10 and Ubuntu LTS 24.04. Although the current version of this module relies on Docker and terminal prompting from a PC and this is not ideal for the use of most of the people with T1D, the *dockerization* of the app facilitates its fast and straightforward integration in a smartphone application, enhancing its potential daily use. The DIY module usage documentation is available in a GitHub public repository (see “Data availability” section).

**Figure 7:**
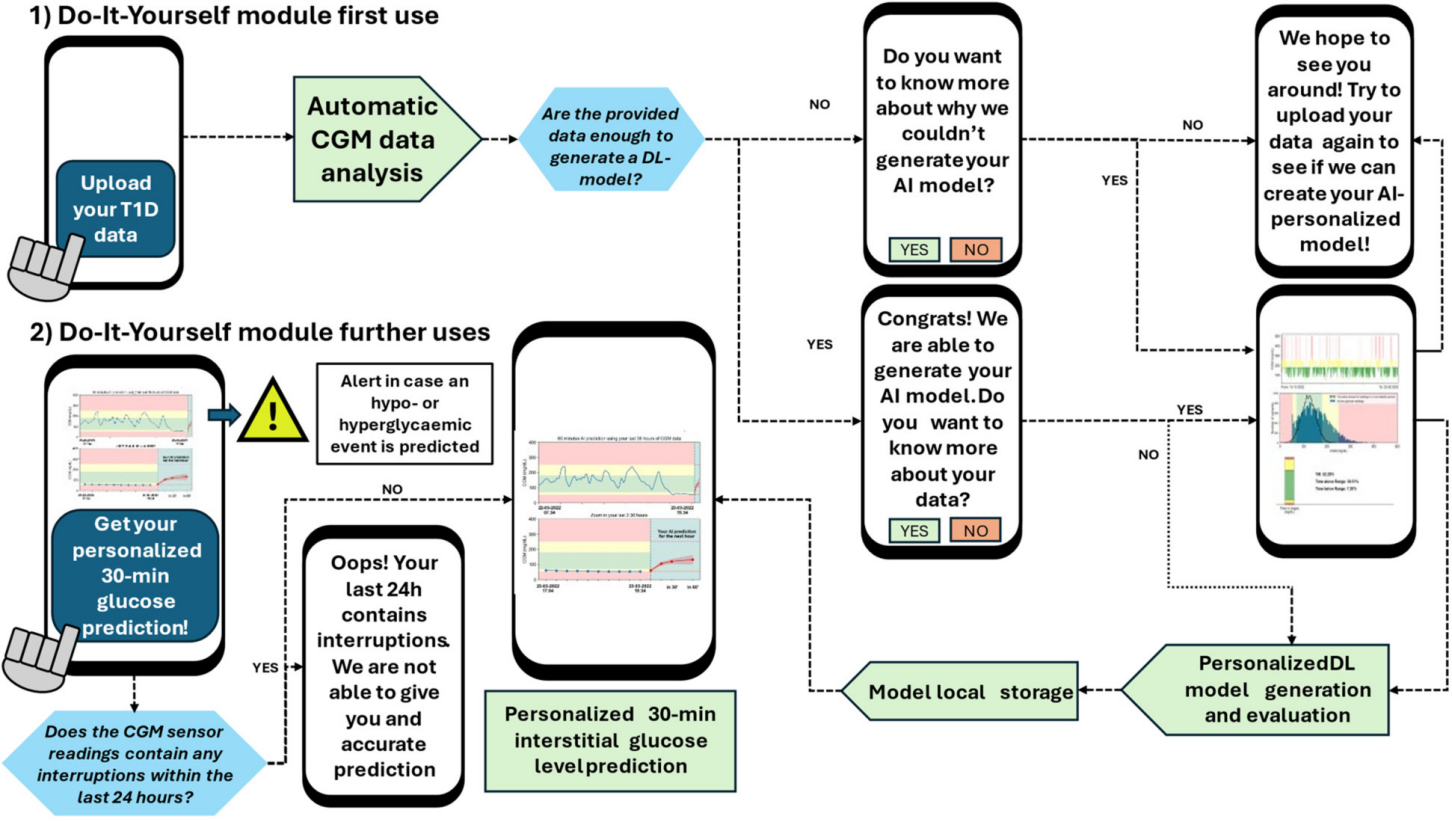
DIY module workflow from the user's perspective. Notice that the first use of the model implies the model generation and evaluation (if enough data is provided). The user's second use only implies the model call and the computation of the 30-min prediction. This workflow considered the scenarios when the user provides or does not have enough data to generate the DL models. Besides, the DIY module prompts the user with messages regarding the provided CGM data, information about the generation (or lack of it) of the AI model, and with alerts if a hypoglycemic of hyperglycemic event is predicted.

For simplicity, only the LSTM architecture was implemented, since it presented the shortest training times with competitive performance. Figure 7 shows the workflow of the DIY module from the user's perspective, in a smartphone, even this module is ready to run on a PC, including the first and subsequent uses, considering the cases when the user provides enough data or not, illustrating the information that is prompted to the user. This design was developed through a co-creation process after carrying out several focus groups that included potential users of this module, and it pursuits user engagement, useful interaction, and information supply regarding their personalized AI models.

## 4 Discussion

To the best of our knowledge, this work has presented, for the first time, a DIY module for personalized AI-based interstitial glucose forecasting for people with T1D using only the CGM user data without involving data from other subjects to train the DL models. Additional data, such as physical activity or carbohydrates intake, was not available in the collected data, but the parametrized design of the module will allow its straightforward inclusion as model inputs. The DL models evaluated in the proposed framework presented similar prediction performance results in terms of RMSE, and the best results in a diabetes-specific metric, *ParkesAB*, when compared to the state-of-the-art, outperforming other LSTM- and CNN-based approaches (24, 26) or modern algorithms designed for time series forecasting, such as N-BEATS (44). Our results were achieved using only CGM data, whereas most of the comparable studies included also insulin and meal intake as input features in their models (24–26, 44). Additionally, 1 year of CGM data was used for the training and validation of the models, being able to capture more seasons-related phenomena compared to other public available standard datasets for benchmarking glucose prediction models, e.g., the Ohio T1DM Dataset (53) which contains only eight weeks. The differences in the duration of the acquired dataset made unfeasible a fair comparison of this framework using this dataset. Unlike most of the previously mentioned studies, the development of the DL models in our study was fully personalized (only data from a single subject was employed to train the algorithms). Moreover, to the best of our knowledge, this is the first work that evaluates acceptable *ISOZone* metric, the most restrictive criteria of ISO 15197:2015 (32), although the proposed DL-models did not fulfil this requirement.

This study also evidenced the need for selecting a diabetes-oriented error metric as a gold standard to compare different glucose prediction models. Results obtained in this work demonstrated that a lower RMSE does not always imply a better prediction in the context of T1D. For example, if a model perfectly predicts all glucose samples that are in range (which are, by far, more than the hypo- and hyper-glycemic samples), it will likely present a lower RMSE than a model that has smaller errors in the critical samples (i.e., hypo- and hyper-glycemic samples), and larger error in the non-critical ones. The latter model will be more clinically useful and safe for this task but would be considered a “worse” model if only RMSE is evaluated. Hence, the use of metrics such as *ISOZone*, *ParkesAB*, or different indexes derived from the diabetic-specific case (26) are pivotal to have an appropriate evaluation of these models (52).

A trimester-wise 4-folds cross-validation was assessed targeting a robust evaluation of the DL models. For each subject (*n* = 29), the models were trained four times, varying the groups of three trimesters used to train the models and the remaining trimester to test them. This approach aimed to demonstrate if, for the proposed architectures, the prediction performance depended on the time of the year used to train and test the DL models. This dependence varied between different subjects, evidencing the burden of the inter-subject variability. Hence, the obtained results suggested that training the models with weeks or even months of data could not be enough, since there are phenomena that might not been considered in the training of the models (habit changes, holidays, periods of increased or decreased activity, etc. that varies over the year). By taking 1 year of data, the negative impact of such intra-subject variability (51) can be potentially diminished. The results obtained using the test set after collecting additional data and training the DL models with 1 year of CGM data support this hypothesis, since the prediction performance slightly improved. Besides, results showed that, with this approach, testing with 1 month of data is representative of the overall performance of the personalized DL models.

A novel loss function, $L_{ISO}$, has been developed to train the DL algorithms. To the best of our knowledge, this is the first work that presents a loss function considering the ISO 15197:2015 standard (32), tailoring the training of the DL models for the interstitial glucose forecasting task. Obtained results are comparable to the standard MSE-based loss function, improving the results in the *ParkesAB* metric for 60-min PH respect to the MSE. Further tuning of $L_{ISO}$ could improve the prediction metric by leading to a more aggressive penalization to the clinically critical errors in glucose prediction. Thus, higher *ParkesAB* and *ISOZone* values could be achieved, potentially outperforming the MSE loss function in the most clinically critical situations of glucose forecasting task. Furthermore, the design of $L_{ISO}$ could have a higher influence on the most vulnerable subjects, i.e., the ones with more hypo- and hyper-glycemic episodes, since it penalizes more the errors for these critical ranges. In this work, no direct relations between prediction performance and the occurrence such episodes were observed (see Supplementary Figure S9; Figures 4, 5).

The inter-subject variability and the dependence of the prediction performance with the available CGM training samples have also been assessed in this work. This is also innovative regarding DL glucose prediction models evaluation. With the proposed LSTM-based and Dil-UNet architectures, no single model has shown optimal prediction performance for all subjects and no statistically significant differences were found between the three architectures. However, in general, the three models showed their best and worst prediction performance with the same subjects regardless of the available training samples. Nonetheless, the comparison of the naïve approach with the proposed architectures allowed to establish a preliminary threshold for both PHs below which was not feasible to generate a reliable personalized DL model. This suggests that inter-subject variability was the main limiting factor to achieve more accurate prediction average results. More complex models specifically designed for time series forecasting, such as N-BEATS (54), or TSMixer (55) could deal with the impact of this phenomena, but it will not remove it.

In this work, it was decided not to include interpolation to deal with the missing data produced by CGM reading interruptions in the training, validation or test sets to assess the model analysis only on real data. Hence, unlike other DL-based approaches to predict glucose concentrations that implemented various types of interpolation when there was a reading interruption within 30 min or 1 h (24–26, 40), the dataset size was significantly decreased in some subjects that contained a considerable number of CGM reading interruptions. The impact of the interpolation can be studied in further work, since the personalized DL-models, especially in those subjects with a number of samples near the threshold, could benefit from a larger number of training and validation samples. Furthermore, in non-personalized models (i.e., that are trained with data from more than one subject), lack of data from one subject can be compensated by others that contribute with more instances. Here, in this work, sensor misfunction or disconnection will lead to the impossibility of a reliable DL model generation.

The DIY module that encapsulates the personalized DL-models was designed considering the daily habits of people with T1D, enhancing personalization and including a subject-oriented models training and evaluation. This was performed supported by a co-creation process through the arrangement of focus groups where potential users of this module offered feedback regarding their needs and preferences of this module. The idea underlying this design was the users' engagement through their empowerment regarding the relationship between AI and their data (30), for example by providing them with information about why their models could (or could not) be generated. Furthermore, it is the user who decides when to be prompted with a glucose prediction or with additional information, thus avoiding the overwhelmingness associated with excessive and non-required information. Its modular design and containerization also facilitate its integration in other applications. However, its integration in smartphone OSs (namely, Android or iOS) is part of future work, to potentially reaching broader populations. In fact, this module is expected to be fully or partly integrated in the WARIFA application (31), as a result of a European project that has designed an application to prevent and manage diverse chronic conditions. As demonstrated in this work, this module is ready to be used standalone in Windows and Ubuntu, as it is already functional and publicly available (see Code Availability). Hence, this DIY module has the potential to have an immediate and positive impact on people who suffer from T1D, although there is more research work to do regarding DL model generation to reach better prediction performance for longer PHs. Additionally, being open source and exhaustively documented, AI researchers and developers could test novel DL architectures that could offer more accurate glucose predictions, use this DIY framework with data from different sensors from the ones employed in this work, integrate this module in an existent application, or enhance the module design from the usability perspective.

Although the DIY paradigm has been validated for T1D, its implementation can extend to other types of diabetes that are using CGM, such as type 2 diabetes (56). Besides, this methodology and the tool itself could be adapted and used for people with different chronic conditions that require continuous monitoring of body signals, and where a computer-aided system can be helpful for clinicians and individuals, such as cardiovascular diseases monitored with wearables devices (57).

Several future opportunities open up after the realization of this work. Firstly, the inclusion of diabetes-related input variables for the DL models, such as insulin, physical activity and carbohydrates intake, that would likely improve prediction performance (44). In this line, the framework might be adapted to generate DL models with less CGM data [e.g., 2 weeks, like the OhioT1DM dataset (53)]. Reducing the monitoring time would likely facilitate the presence of additional diabetes-related variables (e.g., carbohydrate intake, or insulin administration). Additionally, this would allow for easier comparison with other state-of-the-art methods. Related to this, the development of DL architectures that could provide reliable 60-min (and beyond) forecasting should be explored, aiming to increase the positive impact that this tool might have on people's glycemic control. Besides, experiments on parameter tuning of $L_{ISO}$ loss function could be carried out. Obtained results suggest that architectures with more parameters can follow the $L_{ISO}$ penalizations better, but they should be exhaustively studied. Regarding input data, apart from the inclusion of the abovementioned information, prediction performance could significantly benefit from the inclusion of other heterogeneous data, such as glycemic biomarkers, data from electronic health records, etc. Even though these are not real time acquired data, the data fusion paradigm could be explored to evaluate their impact on this task (58). Finally, messages prompted by the DIY module are currently static and equal for all users. However, the potential of the generative AI to personalize not only the CGM prediction, but also the feedback messages provided by this tool, suggest that its implementation on this module will likely increase user's engagement, enhancing our subject-oriented approach (59).

Moreover, if this module is used extensively and constantly over time, model maintenance strategy is a field to be explored, since DL models can lose performance with time (60). Strategies such as transfer learning (47), to re-train the models with new subject's data, or federated learning (61), to employ data from different subjects in the personalized models and to intend a distributed computation of such models, should be studied.

Finally, a pilot study should be carried out to evaluate the real impact of the AI-based DIY module on the daily life of people with T1D. A clinical study also could help to know if this module, as a computer-aid device for the users, will help people to increase their TIR, i.e., to have their glucose concentrations in a healthy range.

## Data Availability

The original contributions presented in the study are publicly available. The code underlying this research can be found here: https://github.com/antorguez95/Personalized-AI-Based-Do-It-Yourself-Glucose-Prediction-tool.
