# Supplementary material for: An AI-based module for interstitial glucose forecasting enabling a “Do-It-Yourself” application for people with type 1 diabetes
Source: Front Digit Health. 2025 Jun 13;7:1534830. doi: 10.3389/fdgth.2025.1534830 (PMC12202434; doi:10.3389/fdgth.2025.1534830)
Supplement: Supplementary file 1 [file Datasheet1.pdf]

Supplementary Material

1 Supplementary Figures

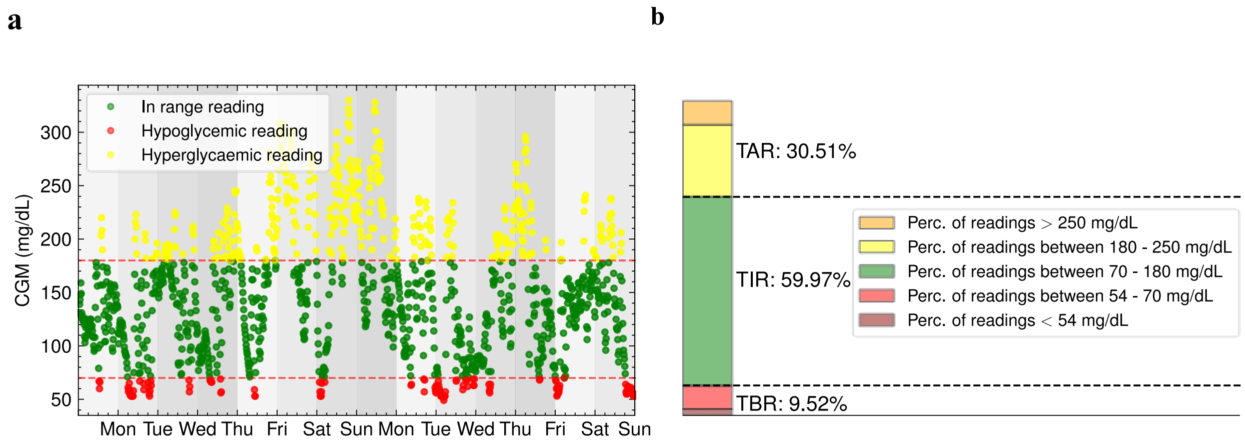

Figure S1. Example of the common visualization of the Continuous Glucose Monitoring (CGM) tool for Type 1 Diabetes (T1D) management. a Segment of a CGM signal for two weeks, where yellow, green, and red dots represent CGM readings in the hyperglycaemic, target, and hypoglycaemic range, respectively. **b** Bar diagram representing the percentage of Time Above Range (TAR), Time In Range (TIR) and Time Below Range (TBR).

a

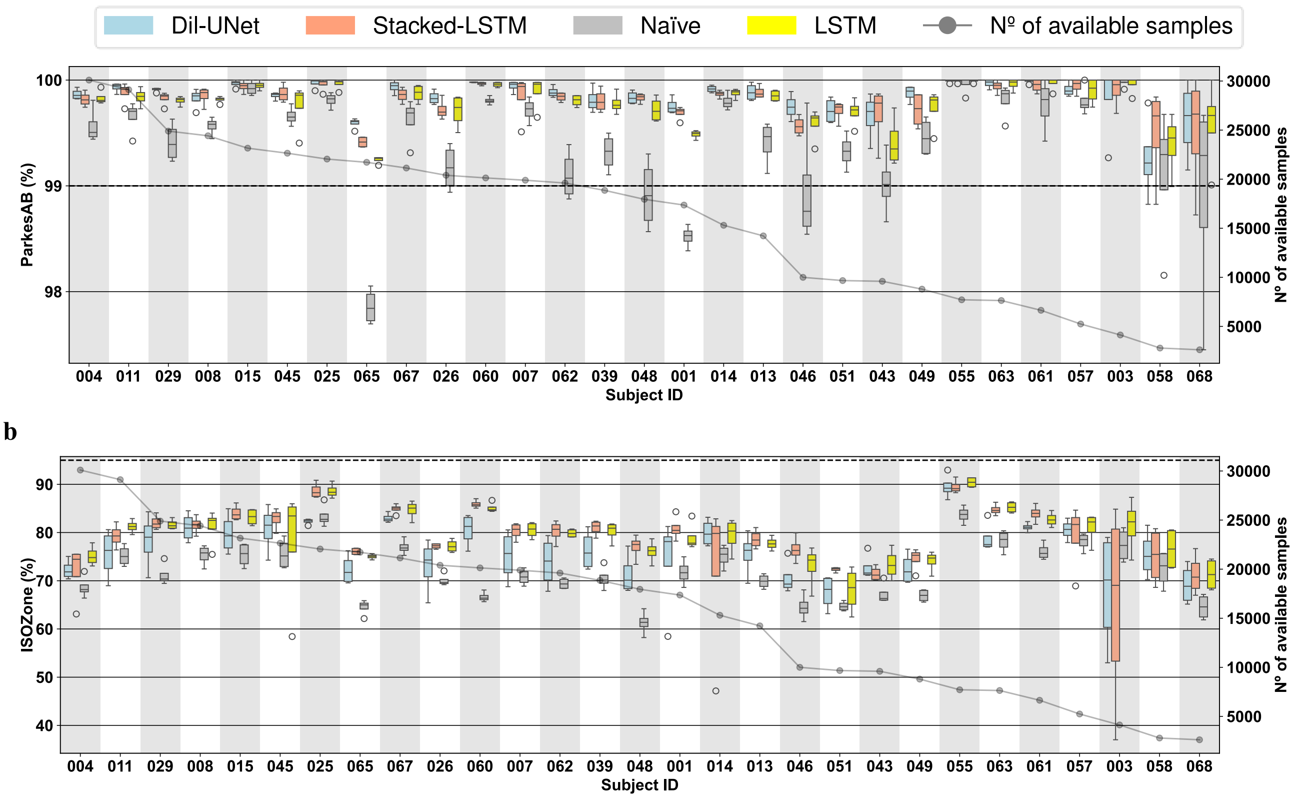

Figure S2. Boxplots representing patient-wise (n = 29) ISO-based prediction metrics (the percentage of the total predicted points within the Parkes acceptable zone (*ParkesAB*) and within the ISO acceptable zone, (*ISOZone*)) computed with the four validation folds (Prediction Horizon (PH) = 30 min) for the four proposed models after being trained with the Mean Squared Error (MSE) loss function. The subjects are sorted in descending order of available instances to train the models. **a** *ParkesAB*. **b** *ISOZone*.

a

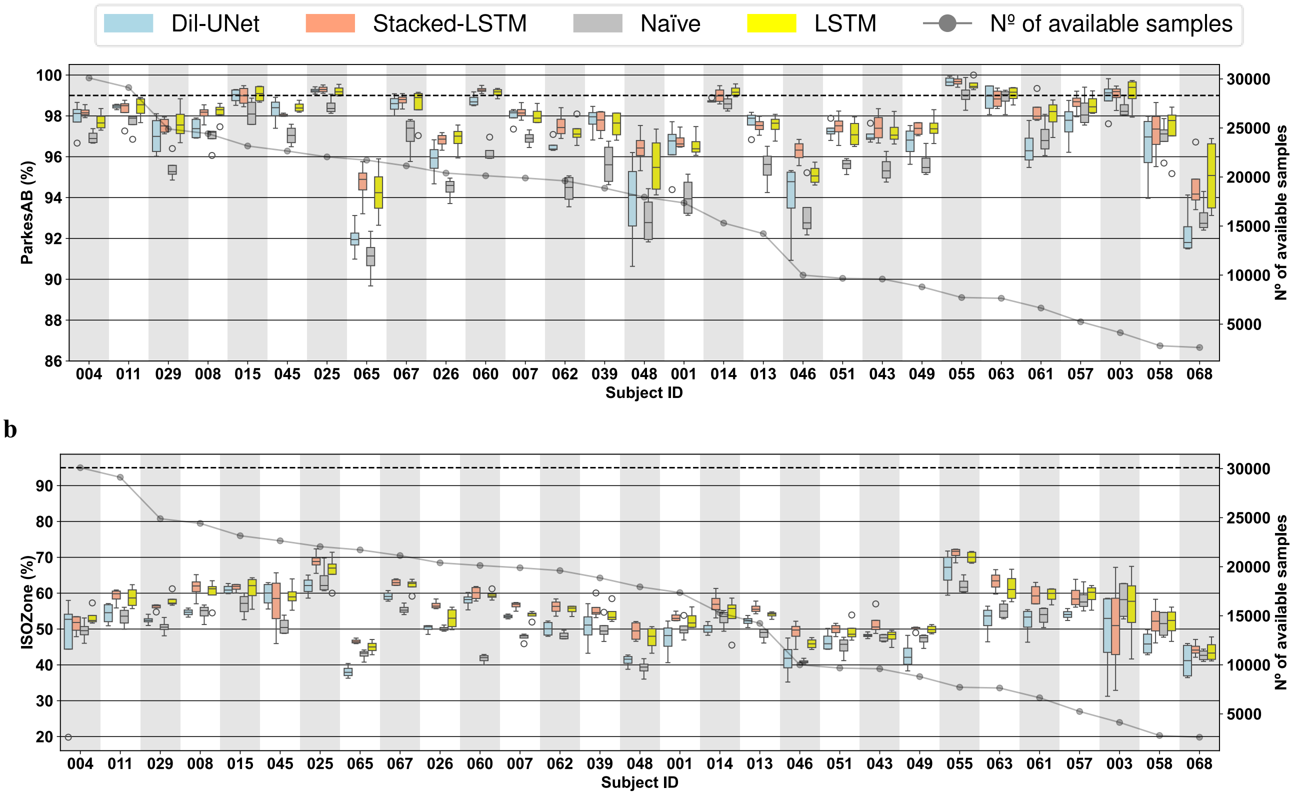

Figure S3. Boxplots representing patient-wise (n = 29) ISO-based prediction metrics (*ParkesAB* and *ISOZone*) computed with the four validation folds (PH = 60 min) for the four proposed models after being trained with the MSE loss function. The subjects are sorted in descending order of available instances to train the models. **a** *ParkesAB*. **b** *ISOZone*.

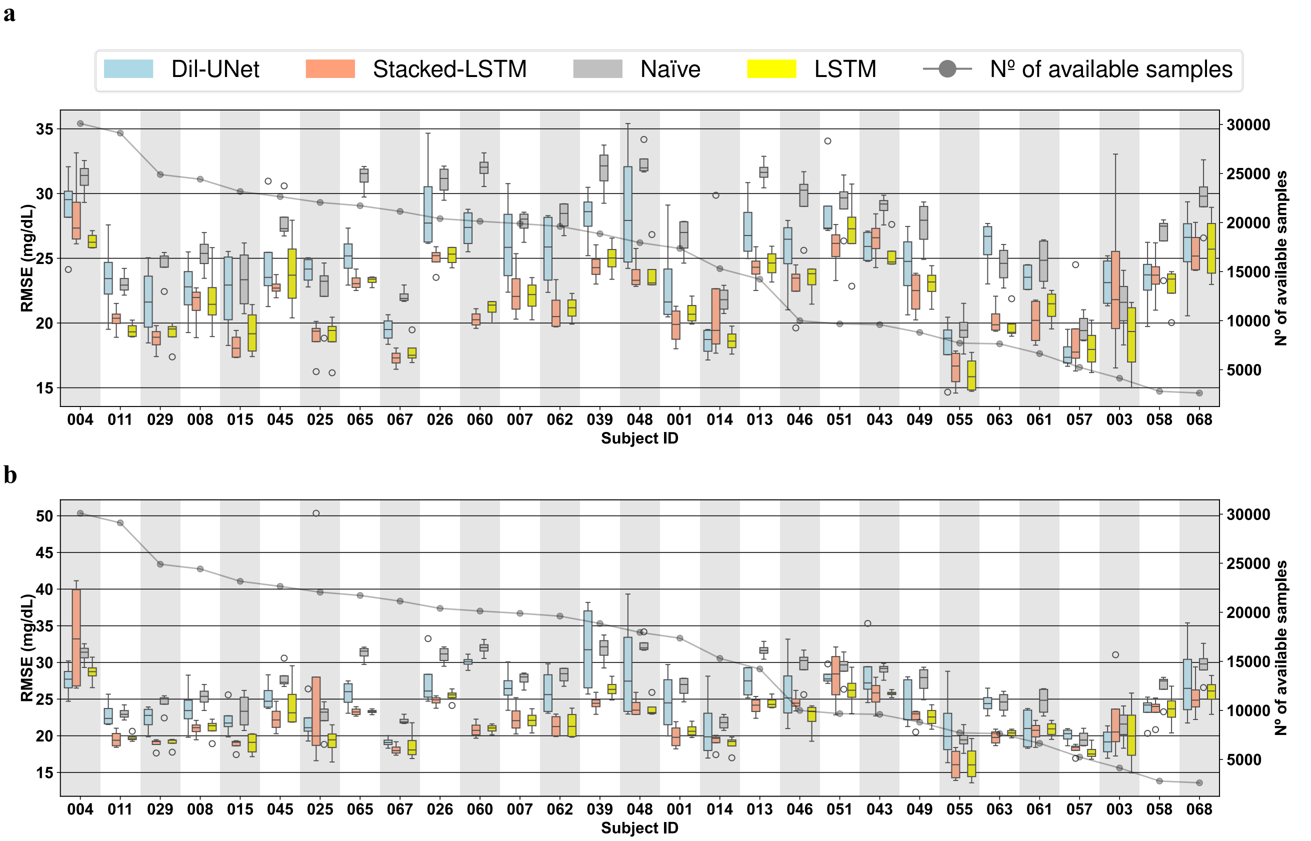

Figure S4. Boxplots representing patient-wise (n = 29) Root Mean Squared Error (RMSE) computed with the four validation folds (PH = 30 min) for the four proposed models. The subjects are sorted in descending order of available instances to train the models. **a** After MSE training. **b** After training with the *ISO-adapted loss function* (L_{ISO}).

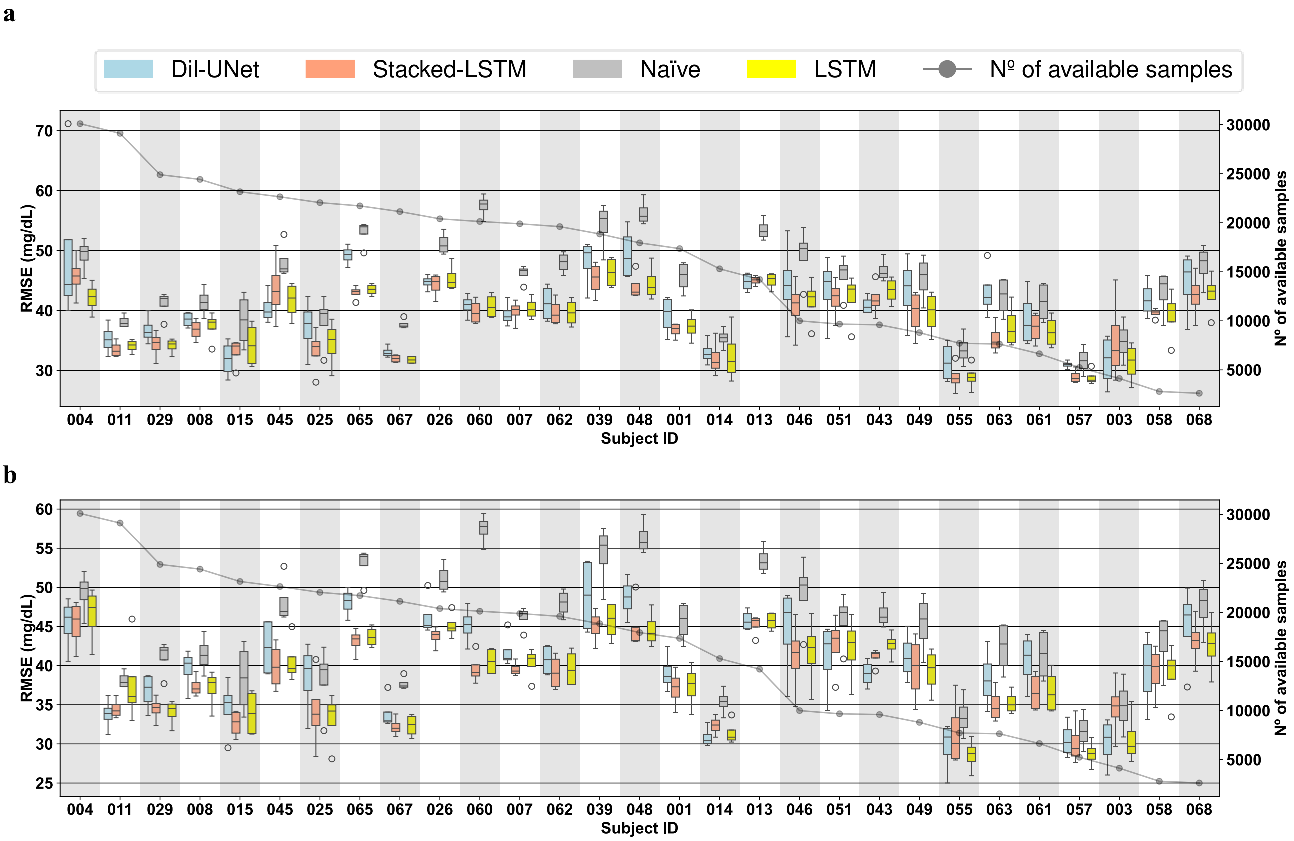

Figure S5. Boxplots representing patient-wise (n = 29) RMSE computed with the four validation folds (PH = 60 min) for the four proposed models. The subjects are sorted in descending order of available instances to train the models. **a** After MSE training. **b** After L_{ISO} training.

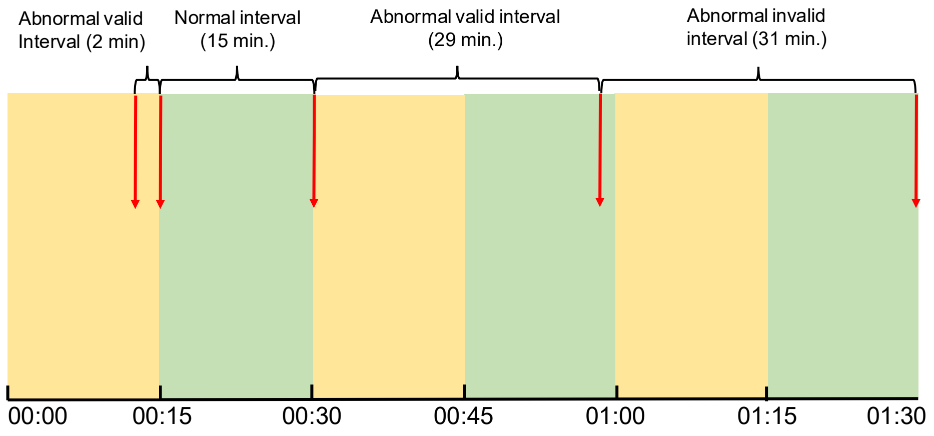

Figure S6. Illustration of valid and invalid time intervals when the sampling period of the CGM sensor is 15 min. Each red arrow represents a sensor reading. An invalid interval is considered an interruption, thus a beginning of a new data block.

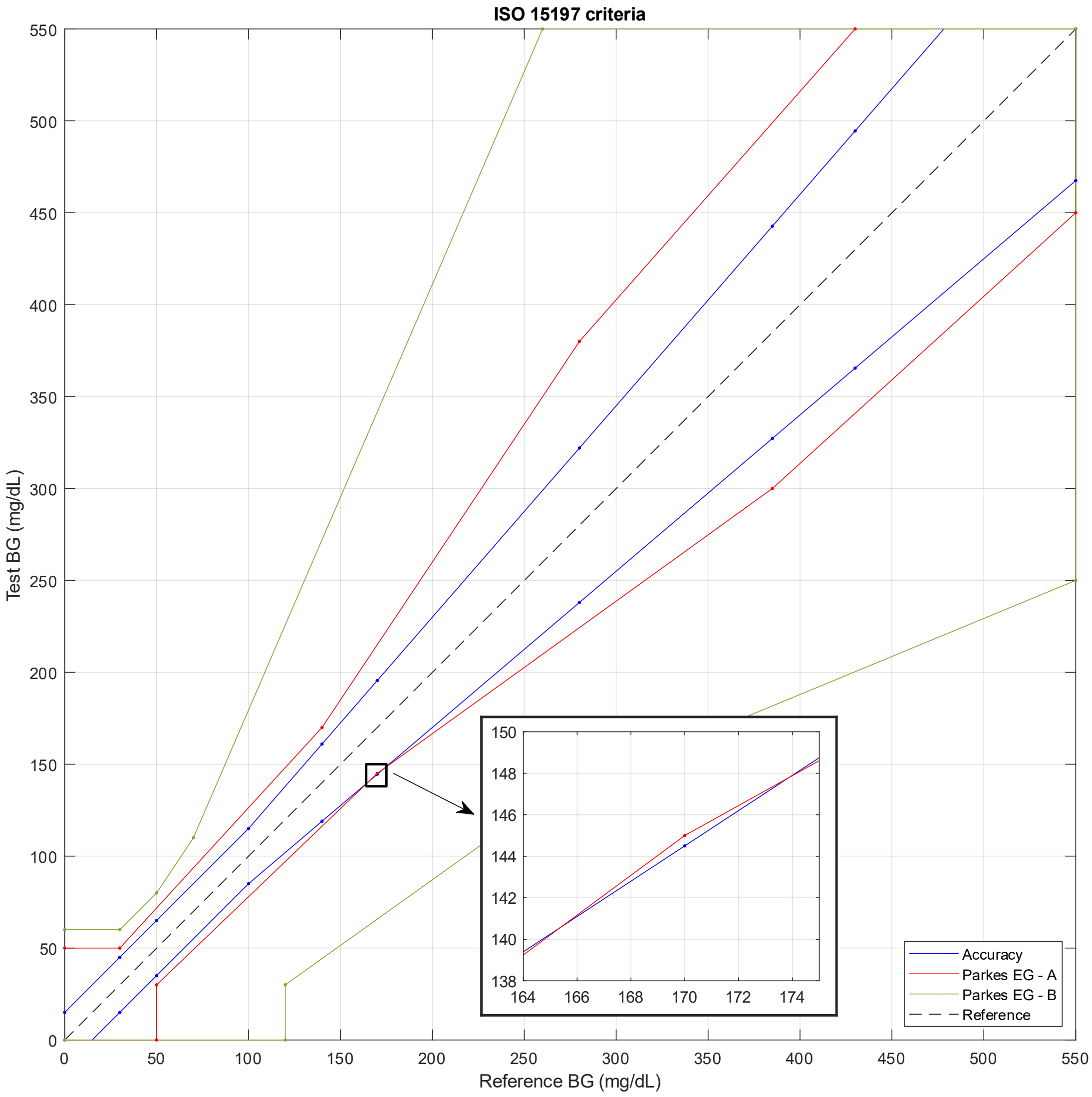

Figure S7. Basis of the development of the L_{ISO} loss function design. a Comparison of maximum admissible error for the accuracy (i.e., *ISOZone*) and Parkes CEG validation criteria included in the ISO 15197:2015 standard.

a

b

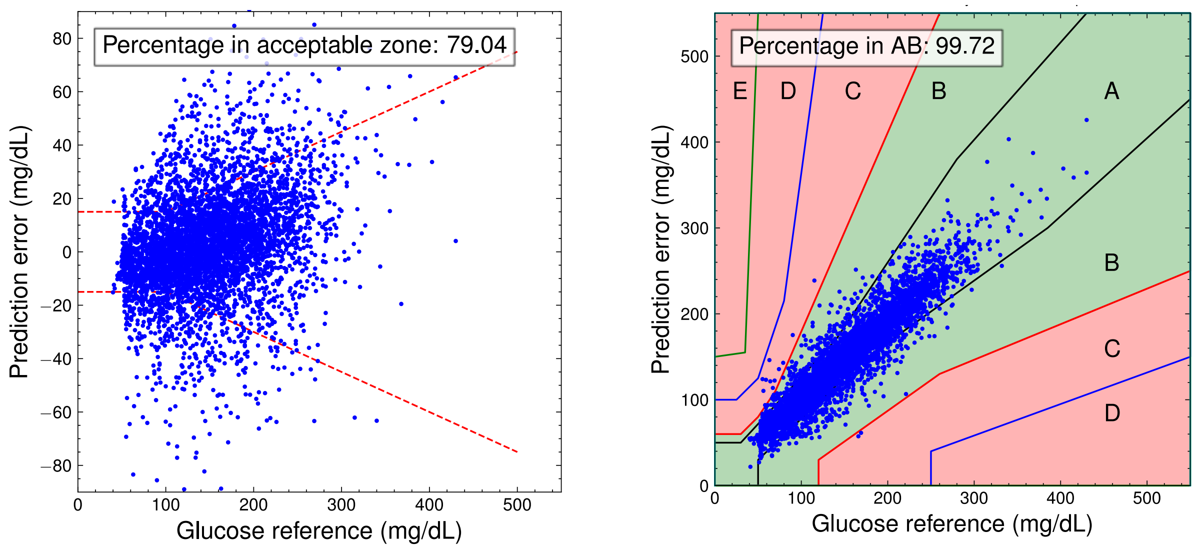

Figure S8. Diagrams of the metrics based on the ISO 15197:2015 standard used in the evaluation of the CGM devices. a *ISOZone* **b** *ParkesAB*.

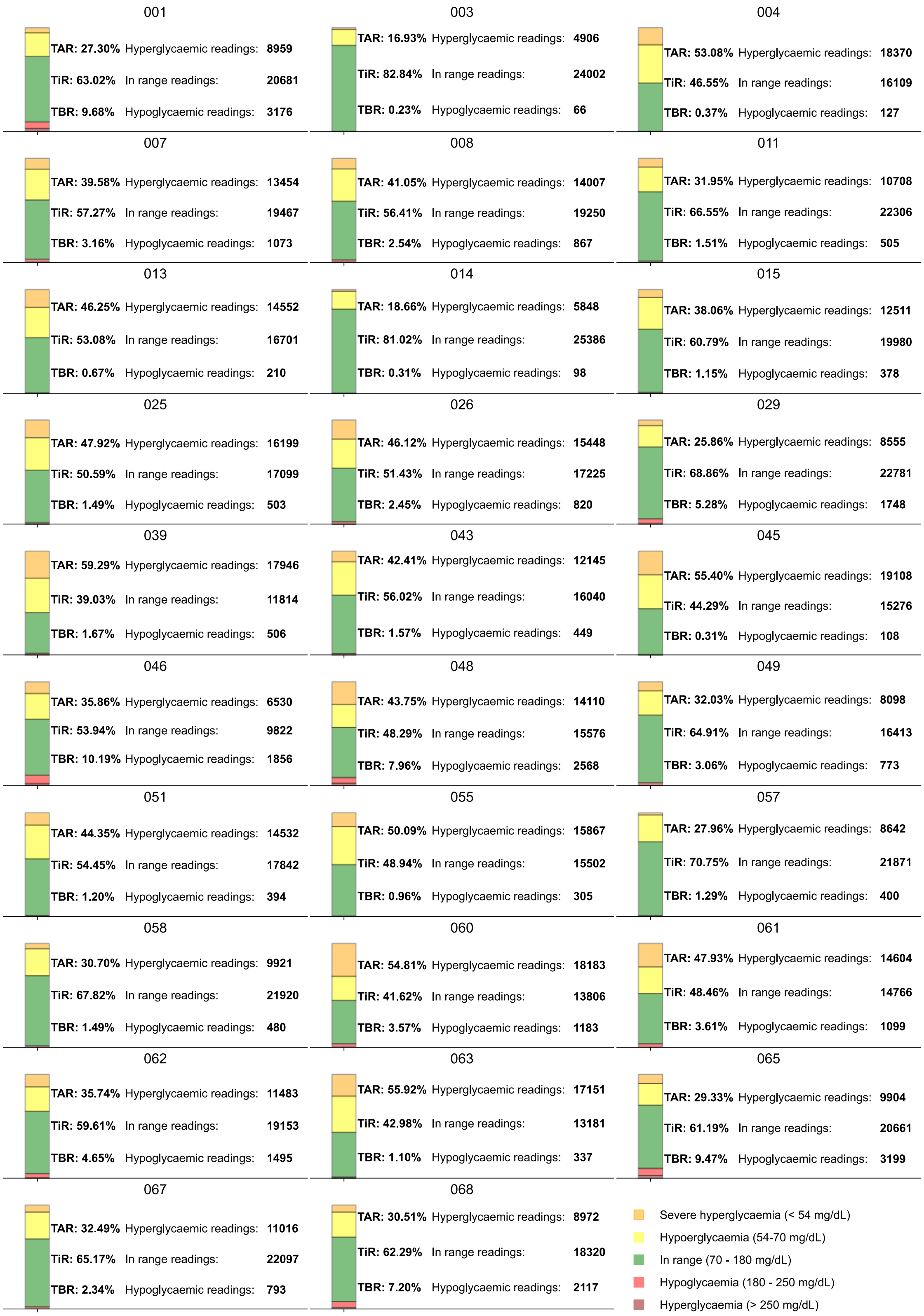

Figure S9. TAR, TIR, and TBR of the available CGM training samples of all included subjects in this work (n = 29).

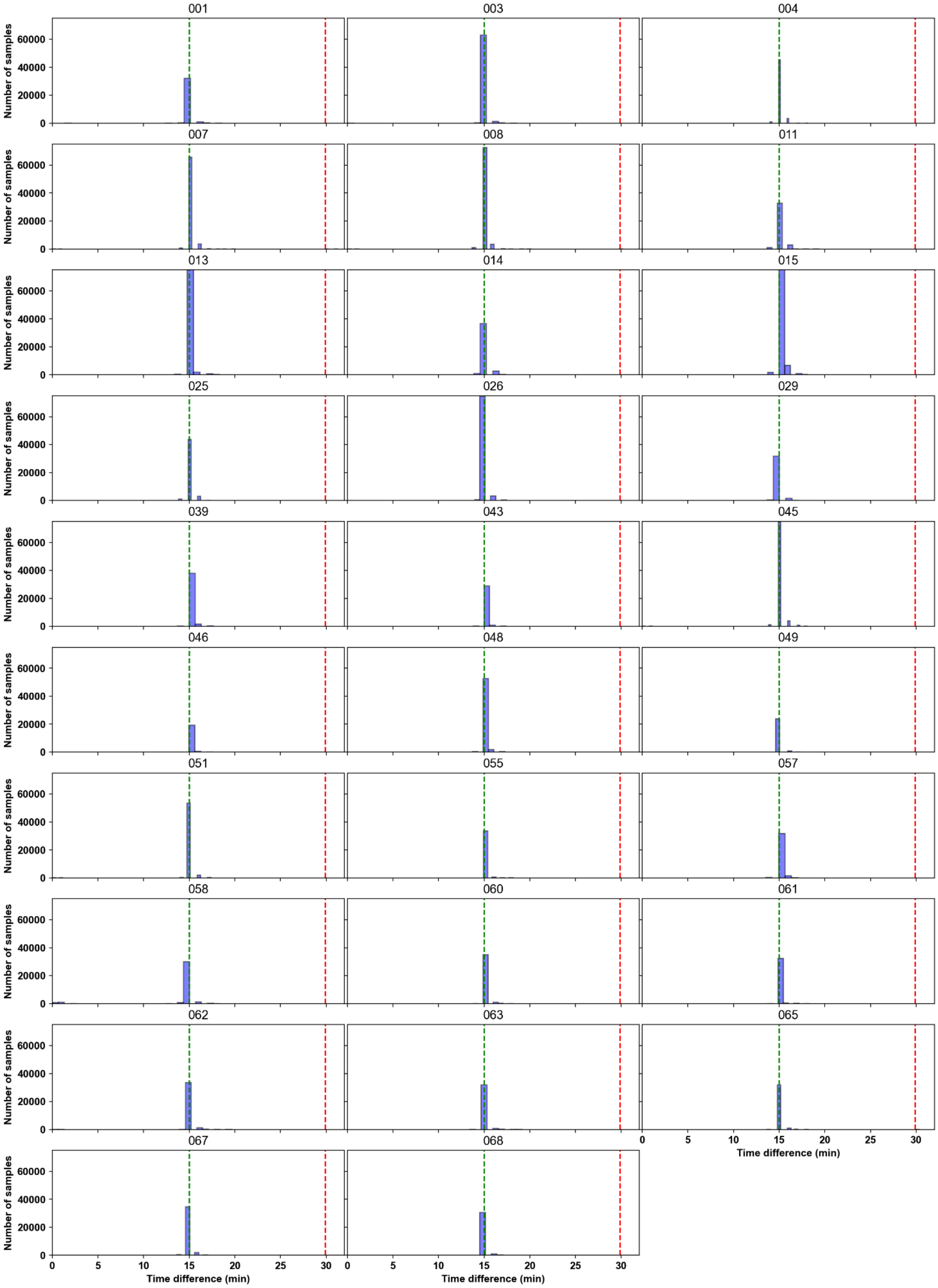

Figure S10. Histogram of the time differences of the consecutive samples of the sensor readings of all included subjects in this work (n = 29). The green dashed line represents the nominal frequency, whereas the red one represent the upper bound of time differences permitted by the proposed framework in the data preparation process.

2 Supplementary Tables

Table S1. Summary of the evaluated Deep Learning (DL) architectures, including model and training hyperparameters, and the total number of parameters for each PH. Notice that, in this work, *N* was fixed to 96.

<table>
<tr><th>DL Architecture</th><th>Model Hyperparameters</th><th>Training Hyperparameters</th><th>#Parameters

(30-min PH / 60-min PH)</th></tr>
<tr><td>LSTM</td><td>Memory cells: N</td><td rowspan="3">Learning rate: 0.0001

Batch size: 1

Epochs: 20</td><td>38210 / 38404</td></tr>
<tr><td>Stacked-LSTM</td><td>Memory cells: [N, N/2, N/2, N/4, N/4]

Drop-out rate: 0.05</td><td>72914 / 72964</td></tr>
<tr><td>DIL-UNet</td><td>Kernel size: 10

Dilation rate: 1

Stride: 1

Drop-out rate: 0.1</td><td>599731 / 599925</td></tr>
</table>

Table S2. Relevant information from all subjects (n=41) for the personalized DL models development, including the recording period times, the different sensor models that used each subject on each period, and the number of CGM samples read by each sensor.

Subject ID	#CGM Samples	Date of the 1st sample data (#Days until last sample)	Sensor Model	#Registered Sensors
**001**	34,602	2022-05-20 (384)	FreeStyle LibreLink	2
	882	2023-06-09 (3)	FreeStyle Libre 3	
**003**	66,203	2021-02-08 (854)	FreeStyle LibreLink	1
**004**	50,319	2022-01-26 (530)	FreeStyle LibreLink	2
	53,984	2020-07-06 (569)	FreeStyle LibreLink	
**007**	71,536	2021-06-01 (769)	FreeStyle LibreLink	1
**008**	26,597	2020-06-08 (289)	FreeStyle LibreLink	2
	78,203	2021-03-25 (837)	FreeStyle LibreLink	
**011**	37,288	2022-05-19 (417)	FreeStyle LibreLink	1
**013**	88,367	2020-06-25 (1,110)	FreeStyle LibreLink	1
**014**	41,079	2021-11-26 (437)	FreeStyle LibreLink	9
	12,588	2023-02-05 (136)	FreeStyle LibreLink	
	55,714	2020-03-10 (626)	FreeStyle LibreLink	
	10,387	2019-12-23 (112)	FreeStyle Libre	
	8,602	2023-06-10 (30)	FreeStyle Libre 3	
	10,093	2016-07-24 (543)	LibreLink	
	8,074	2018-03-13 (643)	FreeStyle LibreLink	
	208	2018-01-21 (21)	FreeStyle LibreLink	
	208	2018-01-21 (21)	LibreLink	
**015**	132,874	2019-08-17 (1,425)	FreeStyle LibreLink	2
	19,697	2022-10-30 (242)	FreeStyle Libre 2	
**019**	33,390	2022-05-16 (360)	FreeStyle LibreLink	2
	5,287	2023-05-16 (57)	FreeStyle LibreLink	
**020**	6,758	2023-06-22 (74)	FreeStyle LibreLink	1
**025**	48,958	2022-01-27 (530)	FreeStyle LibreLink	1
**026**	80,256	2020-06-02 (869)	FreeStyle LibreLink	2
	24,743	2022-10-19 (266)	FreeStyle LibreLink	
**029**	20,653	2022-11-11 (242)	FreeStyle LibreLink	15
	31	2020-07-24 (0)	FreeStyle LibreLink	
	19,978	2021-09-16 (221)	FreeStyle LibreLink	
	1,647	2020-06-24 (17)	FreeStyle LibreLink	
	31	2020-08-01 (0)	FreeStyle LibreLink	
	3,312	2020-06-10 (91)	FreeStyle Libre 2	
	31	2020-07-29 (0)	FreeStyle LibreLink	
	2,982	2020-07-12 (37)	FreeStyle LibreLink	
	No CGM data	-	FreeStyle LibreLink	
	17,872	2022-04-25 (199)	FreeStyle LibreLink	
	31	2020-07-29 (0)	FreeStyle LibreLink	
	134	2020-08-18 (1)	FreeStyle LibreLink	
	34,584	2020-08-31 (381)	FreeStyle LibreLink	
	578	2017-01-02 (6)	LibreLink	
	2,910	2017-01-08 (125)	LibreLink	
**039**	40,996	2021-12-05 (480)	FreeStyle LibreLink	2
	15,295	2021-05-31 (186)	FreeStyle LibreLink	
**042**	7,506	2023-04-20 (82)	FreeStyle LibreLink	1
**043**	30,937	2022-06-15 (391)	FreeStyle LibreLink	1
**044**	7,67	2023-05-18 (12)	FreeStyle LibreLink	4
	227	2023-06-02 (3)	FreeStyle LibreLink	
	2,434	2023-06-08 (30)	FreeStyle LibreLink	
	22,060	2022-02-28 (300)	FreeStyle LibreLink	
**045**	105,697	2020-06-08 (1,128)	FreeStyle LibreLink	1

Table S2. Relevant information from all subjects (n=41) for the personalized DL models development, including the recording period times, the different sensor models that used each subject on each period, and the number of CGM samples read by each sensor.

**046**	20,808	2022-04-28 (393)	FreeStyle LibreLink	4
	19,991	2020-07-28 (406)	FreeStyle LibreLink	
	4,326	2022-02-05 (82)	FreeStyle LibreLink	
	2,421	2021-09-08 (147)	FreeStyle LibreLink	
**047**	12,115	2022-06-04 (145)	FreeStyle LibreLink	4
	111	2021-12-04 (3)	FreeStyle LibreLink	
	15,196	2021-11-16 (200)	FreeStyle LibreLink	
	20,635	2022-11-09 (244)	FreeStyle LibreLink	
**048**	801	2022-09-22 (17)	FreeStyle LibreLink	2
	56,253	2020-12-14 (646)	FreeStyle LibreLink	
**049**	25,442	2020-11-27 (367)	FreeStyle LibreLink	1
**051**	57,363	2021-10-09 (641)	FreeStyle LibreLink	2
	26,152	2020-11-27 (313)	FreeStyle LibreLink	
**052**	No CGM data	-	FreeStyle Libre 3	12
	25,732	2023-05-19 (30)	FreeStyle Libre 3	
	645	2023-05-19 (2)	FreeStyle Libre 3	
	14,798	2022-10-25 (206)	FreeStyle LibreLink	
	910	2022-07-23 (10)	FreeStyle LibreLink	
	621	2023-05-19 (44)	FreeStyle Libre 3	
	1,325	2023-06-18 (14)	FreeStyle LibreLink	
	7,243	2022-08-02 (85)	FreeStyle LibreLink	
	10,715	2023-07-02 (63)	FreeStyle Libre 3	
	2,392	2023-07-17 (26)	FreeStyle LibreLink	
	No CGM data	-	FreeStyle LibreLink	
	No CGM data	-	FreeStyle Libre 3	
**053**	6,902	2023-06-23 (73)	FreeStyle LibreLink	1
**054**	17,169	2023-02-13 (203)	FreeStyle LibreLink	7
	No CGM data	-	FreeStyle LibreLink	
	No CGM data	-	FreeStyle LibreLink	
	4,908	2022-12-13 (58)	FreeStyle LibreLink	
	No CGM data	-	FreeStyle LibreLink	
	62	2022-11-13 (7)	FreeStyle LibreLink	
	5,829	2022-09-12 (91)	FreeStyle LibreLink	
**055**	35,292	2022-07-24 (407)	FreeStyle LibreLink	1
**056**	20,887	2022-10-25 (301)	FreeStyle LibreLink	1
**057**	34,336	2022-07-24 (407)	FreeStyle LibreLink	1
**058**	35,938	2022-07-27 (403)	FreeStyle LibreLink	1
**059**	2,893	2023-01-30 (10)	FreeStyle Libre 3	2
	9,276	2023-07-06 (50)	FreeStyle Libre 3	
**060**	37,011	2022-07-24 (407)	FreeStyle LibreLink	1
**061**	34,209	2022-07-24 (407)	FreeStyle LibreLink	1
**062**	36,085	2022-07-24 (408)	FreeStyle LibreLink	1
**063**	33,889	2022-07-22 (409)	FreeStyle LibreLink	1
**064**	31	2022-09-05 (0)	FreeStyle LibreLink	1
	31	2022-09-05 (0)	FreeStyle LibreLink	
	4,797	2022-09-05 (52)	FreeStyle LibreLink	
	31	2022-09-05 (0)	FreeStyle LibreLink	
**065**	33,829	2021-09-29 (366)	FreeStyle LibreLink	1
**066**	11,478	2022-03-10 (125)	FreeStyle LibreLink	2
	14,683	2022-12-10 (260)	FreeStyle Libre 2	
**067**	38,036	2022-07-24 (409)	FreeStyle LibreLink	1
**068**	32,900	2022-07-24 (409)	FreeStyle LibreLink	1

Table S3. Test results of RMSE, *ParkesAB*, and *ISOZone* metrics of the proposed DL models after training them with one year of CGM data using L_{ISO}loss function when the test set included 30, 90, 180, and 365 days for 30-min PH. Results are shown as mean±Standard Deviation (SD) of the included subjects. Results in bold font indicate the model with the best performance for a specific metric.

Test days = 30, n = 20			
Metric / Model	RMSE (mg/dL)	*ParkesAB* (%)	*ISOZone* (%)
**LSTM**	**20.85±4.11**	**99.81±0.36**	**81.85±7.02**
**Stacked-LSTM**	21.59±3.80	99.80±0.32	80.71±5.71
**Dil-Unet**	25.74±5.40	99.79±0.31	74.18±9.19
**Test days = 90, n = 20**			
Metric / Model	RMSE (mg/dL)	*ParkesAB* (%)	*ISOZone* (%)
**LSTM**	**20.38±3.13**	99.84±0.23	**82.02±5.72**
**Stacked-LSTM**	21.44±2.82	99.85±0.20	80.29±5.09
**Dil-Unet**	25.04±5.51	**99.87±0.21**	75.05±8.79
**Test days = 180, n = 20**			
Metric / Model	RMSE (mg/dL)	*ParkesAB* (%)	*ISOZone* (%)
**LSTM**	**20.82±3.21**	99.81±0.22	**81.54±5.63**
**Stacked-LSTM**	21.91±2.96	99.82±0.19	79.77±5.00
**Dil-Unet**	24.95±5.41	**99.86±0.15**	75.74±8.22
**Test days = 365, n = 20**			
Metric / Model	RMSE (mg/dL)	*ParkesAB* (%)	*ISOZone* (%)
**LSTM**	**20.71±3.59**	99.80±0.24	**80.78±8.32**
**Stacked-LSTM**	22.34±3.31	99.82±0.17	78.57±6.34
**Dil-Unet**	23.73±3.21	**99.85±0.16**	78.16±6.60

Table S4. Test results of RMSE, *ParkesAB*, and *ISOZone* metrics of the proposed DL models after training them with one year of CGM data using L_{ISO}loss function when the test set included 30, 90, 180, and 365 days for 60-min PH. Results are shown as mean±SD of the included subjects. Results in bold font indicate the model with the best performance for a specific metric.

Model \ Metric	RMSE (mg/dL)	*ParkesAB* (%)	*ISOZone* (%)
**Test days = 30, n = 20**			
**LSTM**	37.28±6.84	98.17±1.73	**58.64±7.93**
**Stacked-LSTM**	**36.65±6.58**	**98.26±1.64**	58.61±8.76
**Dil-Unet**	38.90±6.87	97.81±2.45	54.70±8.44
**Test days = 90, n = 20**			
**LSTM**	36.65±5.82	**98.22±1.55**	**58.86±6.73**
**Stacked-LSTM**	**36.45±5.68**	98.18±1.56	58.56±7.75
**Dil-Unet**	37.90±5.49	97.90±2.28	55.13±6.33
**Test days = 180, n = 20**			
**LSTM**	37.04±5.46	**98.13±1.40**	**58.72±6.58**
**Stacked-LSTM**	36.90±5.53	98.07±1.51	58.35±7.12
**Dil-Unet**	**38.16±5.22**	97.84±1.64	54.93±6.00
**Test days = 365, n = 20**			
**LSTM**	36.80±7.03	**98.11±1.64**	59.84±8.85
**Stacked-LSTM**	**36.14±6.14**	**98.11±1.68**	**60.51±8.72**
**Dil-Unet**	37.42±5.17	97.99±1.96	56.10±7.77
